# Photothermal therapy of copper incorporated nanomaterials for biomedicine

**DOI:** 10.1186/s40824-023-00461-z

**Published:** 2023-11-24

**Authors:** Rong Wang, Ziwei Huang, Yunxiao Xiao, Tao Huang, Jie Ming

**Affiliations:** Department of Breast and Thyroid Surgery, Union Hospital, Tongji Medical College, Huazhong University of Science and Technology, 1277 Jiefang Avenue, Wuhan, 430022 People’s Republic of China

**Keywords:** Copper, Photothermal therapy, Nanomaterials, Antitumor, Tissue regeneration

## Abstract

Studies have reported on the significance of copper incorporated nanomaterials (CINMs) in cancer theranostics and tissue regeneration. Given their unique physicochemical properties and tunable nanostructures, CINMs are used in photothermal therapy (PTT) and photothermal-derived combination therapies. They have the potential to overcome the challenges of unsatisfactory efficacy of conventional therapies in an efficient and non-invasive manner. This review summarizes the recent advances in CINMs-based PTT in biomedicine. First, the classification and structure of CINMs are introduced. CINMs-based PTT combination therapy in tumors and PTT guided by multiple imaging modalities are then reviewed. Various representative designs of CINMs-based PTT in bone, skin and other organs are presented. Furthermore, the biosafety of CINMs is discussed. Finally, this analysis delves into the current challenges that researchers face and offers an optimistic outlook on the prospects of clinical translational research in this field. This review aims at elucidating on the applications of CINMs-based PTT and derived combination therapies in biomedicine to encourage future design and clinical translation.

**Graphical Abstract:**

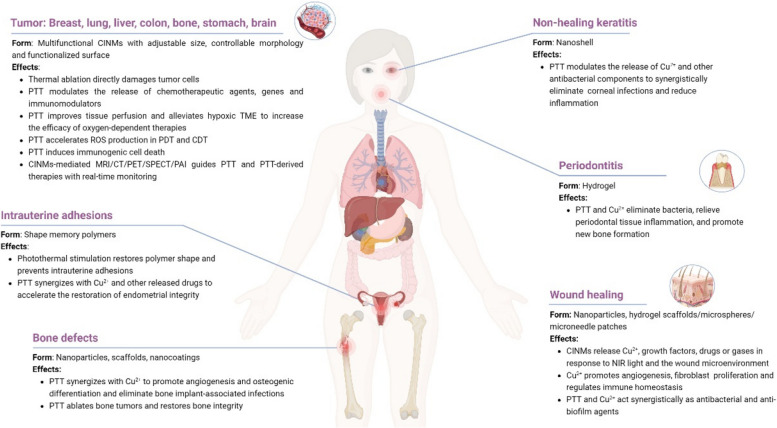

## Introduction

Globally, cancer incidences and mortality rates are rapidly increasing [1]. Traditional treatment options, such as surgery, chemotherapy, and radiotherapy are limited by low efficacies, drug resistance and significant side effects [2, 3]. Chronic skin wound healing and bone defect repair are common clinical challenges [4, 5]. Rapid advances in nanomedicine have facilitated extensive research in multifunctional light-induced nanoplatforms for cancer and tissue regeneration [6, 7]. Photothermal agents (PTAs) irradiated by specific light wavelengths can induce local hyperthermia by absorbing photon energy and converting the increased kinetic energy into thermal energy. This process, known as photothermal therapy (PTT), is a highly effective and non-invasive treatment method that can induce cancer cell or pathogenic bacterial death [8–10]. Moreover, PTT can facilitate other therapies by improving tissue perfusion and enhancing cell membrane permeability. These effects can enhance the overall therapeutic effects and overcome the limitations associated with single treatment approaches [11, 12]. Various synergistic therapies have been developed by combining PTT with other modalities, such as chemotherapy, chemodynamic therapy (CDT), and photodynamic therapy (PDT) [13–16].

Compared with other noble metal nanoparticles, copper nanoparticles have gained significant attention due to their high natural content and cost-effectiveness [17]. As an indispensable trace element in the human body, copper is directly involved in a variety of biological processes and not only promotes angiogenesis and wound healing, but also has significant antibacterial advantages [18]. Copper incorporated nanomaterials (CINMs) have an intense and tunable localized surface plasmon resonance (LSPR) in the near-infrared (NIR) biological window, which brings excellent photothermal conversion efficiency (PCE) for PTT and photoacoustic imaging (PAI) [19–22]. CINMs have good catalytic properties and mediate Fenton-like reactions more efficiently than iron-based nanomaterials under a wide range of pH conditions [23]. CINMs can also function as photosensitizers to induce bacterial and tumor cell death via PDT [24, 25]. The optical properties and catalytic activities of CINMs can be improved by adjusting their shapes, sizes and composition [17]. Given these properties, the significance of CINMs has been investigated in various biomedical applications, such as tumor imaging and treatment, and tissue regeneration [26–31]. As an excellent candidate for personalized nanomedicines, CINMs can provide a platform for combination of multiple therapeutic and diagnostic modalities to visualize synergistic therapeutic effects. However, it is undeniable that the oxidizing tendency of copper under atmospheric conditions limits the preparation of copper nanoparticles. The agglomeration of copper nanoparticles may reduce the specific surface area, and the stability during catalysis is not satisfactory, which may affect the catalytic activity [17, 29]. The dose-dependent cytotoxicity may limit the application of CINMs in biomedical fields [32, 33]. All these issues need more attention in the future.

**Fig. 1:**
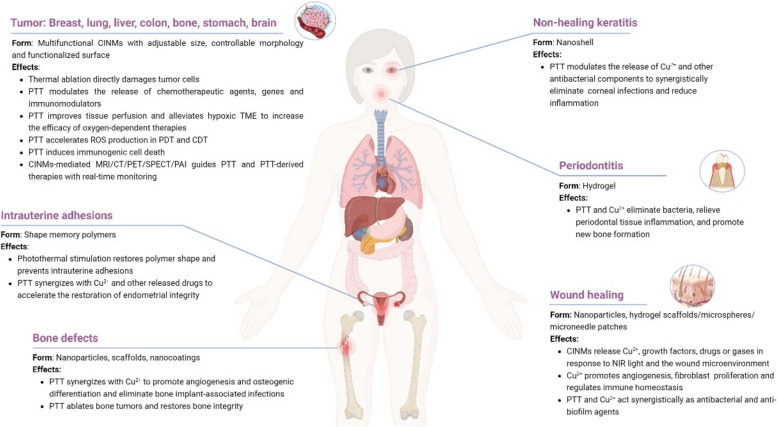
Schematic diagram of CINMs-based PTT in antitumor and tissue regeneration applications

Several reviews on CINMs have been published, but few systematic and comprehensive summaries are available [34–38]. Considering the rapid development of CINMs in the biomedical field, we report on recent advances in photothermal-derived combination therapies of CINMs for cancer therapy, cancer imaging, and tissue regeneration in this review (Tables 1 and 2, and Fig. 1). This review begins with an overview of the classification and structure of CINMs, followed by representative studies of various CINMs-based photothermal combination therapies in cancer therapy and imaging, and a discussion of the current problems of the various therapies. The applications of CINMs-based PTT in tissue regeneration, such as skin and bone, are then summarized. Moreover, the biosafety of CINMs is discussed. Finally, the current challenges, possible solutions and future prospects for clinical translational research are considered. This review aims at elucidating the applications of PTT-derived combination therapies of CINMs in biomedicine and to encourage future designs and clinical translation.

**Table 1:** Applications of CINMs-based PTT in cancer therapy and imaging

Nanomaterial	Therapeutic remarks	Laser parameters	Tumor model	Reference
IPNs	PTT/chemotherapy/PDT	980 nm, 2.0 W·cm^−2^, 15 min	4T1 tumor	[14]
Cu_2_O@CaCO_3_@HA (CCH)	PTT/PDT/CDT/immunotherapy	1064 nm, 0.5 W·cm^−2^, 5 min	CT26.WT tumor	[15]
LDH-CuS	PTT/CDT/PDT/PAI/lysosome-targeting	808 nm, 1.5 W·cm^−2^, 5 min	4T1 tumor	[39]
Gox@CuS	PTT/CDT/PDT/starvation therapy	808 nm, 5.0 W·cm^−2^, 10 min	B16F10 tumor	[40]
TRF-mCuGd	PTT/CDT/MRI	808 nm, 0.8 W·cm^−2^, 10 min	MDA-MB-231 and MCF-7/DDP tumors	[27]
Cu_3_BiS_3_ nanorods	PTT/radiotherapy/PAI/CT	1064 nm, 1.0 W·cm^−2^, 6 min	4T1 tumor	[41]
Cu_2_MoS_4_ (CMS)/Au	PTT/PDT/immunotherapy/CT/PAI	808 nm, 0.5 W·cm^−2^, 5 min	U14 tumor	[19]
BCGCR	PTT/CDT/MRI/NIRF	980 nm, 0.8 W·cm^−2^, 10 min	U87MG tumor	[42]
HMSNs@PDA-Cu	PTT/CDT	808 nm, 0.6 W·cm^−2^, 3 min	MCF-7 tumor	[43]
ICG/Cu-LDH@BSA-DOX	PTT/PDT/chemotherapy	808 nm, 0.3 W·cm^−2^, 2 min	B16F0 tumor	[44]
HMON@CuS/Gd	PTT/PDT/MRI/fluorescence	808 nm, 0.8 W·cm^−2^, 8 min	HGC-27 tumor	[45]
CuS NPs-PEG-Mal	PTT/immunotherapy	808 nm, 0.45 W·cm^−2^, 5 min	4T1 tumor	[46]
FA-CD@PP-CpG	PTT/PDT/chemotherapy/immunotherapy	650 nm, 4.5 mW·cm^−2^, 5 min for PDT; 808 nm, 0.987 W·cm^−2^, 5 min for PTT	4T1 tumor	[47]
CuS@OVA-PLGA-NPs	PTT/immunotherapy	980 nm, 1.0 W·cm^−2^, 10 min	4T1 tumor	[48]
CuS-SF@CMV	PTT/immunotherapy/chemotherapy	808 nm, 0.6 W·cm^−2^, 5 min	H22 tumor	[49]
Cu_2_MnS_2_ NPs	PTT/MRI/MSOT	1064 nm, 0.6 W·cm^−2^, 10 min	S180 tumor	[50]
Cu_2_ZnSnS_4_ (CZTS)@BSA	PTT/MRI/PAI	808 nm, 1.0 W·cm^−2^, 5 min	H22 tumor	[51]
Cu_2-x_Se-Au Janus NPs	PTT/CDT/photocatalytic therapy/CT/PAI	808 nm, 0.36 W·cm^−2^, 10 min	4T1 tumor	[52]
C-m-ABs	PTT/PDT/CDT/chemotherapy/MRI/PA/NIRF	808 nm, 1.4 W·cm^−2^, 8 min	MGC-803 tumor	[53]
Au@Cu_2_O	PTT/PAI	808 nm, 1.0 W·cm^−2^, 5 min	HCT tumor	[22]
Core–satellite nanoconstructs (CSNC)	PTT/PDT/PET/fluorescence/Cerenkov luminescence/Cerenkov radiation energy transfer	980 nm, 4.0 W·cm^−2^, 10 min for PTT; 660 nm, 0.05 W·cm^−2^, 20 min for PDT	4T1 tumor	[54]
BPMN-CuS/DOX	PTT/chemotherapy/CDT	808 nm, 1.0 W·cm^−2^, 3 min	B16F10 tumor	[55]
PZTC/SS/HA	PTT/CDT	1064 nm, 1.0 W·cm^−2^, 10 min	HCT-116 tumor	[56]
Cu-BTC@PDA	PTT/CDT	808 nm, 1.0 W·cm^−2^, 10 min	B16F10 tumor	[57]
CAL@PG NPs	PTT/chemotherapy/CDT	1064 nm, 1.35 W·cm^−2^, 10 min	MHCC97H tumor	[58]
Cu(II)/LRu/PDA NPs	PTT/PDT/MRI/photoacoustic tomography	808 nm, 1.0 W·cm^−2^, 12 min for PTT; 660 nm, 1.0 W·cm^−2^, 12 min for PDT	HeLa tumor	[59]

**Table 2:** Applications of CINMs-based PTT in tissue regeneration

Nanomaterial	Therapeutic remarks	Laser parameters	Bacteria/tumor model	Performance	Reference
CS-PLA/PCL membranes	PTT/release of Cu^2+^/patterned membranes	808 nm, 0.4 W·cm^−2^, 15 min	B16F10 tumor; diabetic wound	Antitumor and wound healing	[31]
PATA-C4@CuS	PTT/PDT/bacteria-targeting	980 nm, 1.5 W·cm^−2^, 3 min	Bacteria-infected wound	Antibacterial and wound healing	[60]
CuS NDs	PTT/release of Cu^2+^	808 nm, 2.5 W·cm^−2^, 1 min	MRSA-infected diabetic wound	Antibacterial and wound healing	[61]
Cu_3_SnS_4_ nanoflakes	PTT/PDT/release of Cu^2+^/bacteria-targeting/active SERS imaging substrate	808 nm, 1.0 W·cm^−2^, 10 min	MRSA-infected wound	Antibacterial, wound healing and bacteria detection in vitro	[62]
BG-CFS scaffolds	PTT/release of Ca^2+^, SiO_4_^4−^, PO_4_^3−^, Cu^+^, Se^2+^ and Fe^3+^	808 nm, 0.55 W·cm^−2^, 10 min	Saos-2 bone tumor; femoral defect	Antitumor and bone reconstruction	[63]
Cu-DCA NZs	PTT/relief of hypoxia/release of Cu^2+^	808 nm, 1.0 W·cm^−2^, 5 min	*Staphylococcus aureus (S. aureus)*-infected diabetic wound	Antibacterial and wound healing	[64]

## The classification and structure of CINMs

Many CINMs have been reported for biomedical applications, mainly including copper oxides, copper-based chalcogenides, copper nanoalloys, and copper-incorporated nanocomposites. Copper oxides include CuO and Cu_2_O, which are p-type semiconductor and have been widely used in batteries, gas sensors, and catalysis [65]. In the biomedical field, copper oxides can induce oxidative stress to play a tumor-killing role, and can also catalyze the generation of oxygen from endogenous hydrogen peroxide (H_2_O_2_) to alleviate tumor hypoxia [32, 66, 67]. Moreover, copper oxides have potent antibacterial activity and potential to promote wound healing [68]. The compositional, structural, and stoichiometric diversity of copper chalcogenides endows them with excellent electrical, optical, and magnetic properties, and they show great potential for energy conversion, energy storage, and biomedical applications [69, 70]. Binary copper chalcogenides, including Cu_2-x_S, Cu_2-x_Se, Cu_2-x_Te (0 ≤ x ≤ 1), are widely used in photothermal therapy and biomedical imaging due to the tunable LSPR [23, 52, 70–73]. Of these, the most versatile copper sulfide has been used for tumor imaging and therapy, antibacterial, and tissue regeneration [74–76]. Ternary and quaternary copper chalcogenides can be prepared by introducing the main-group metals (Sn, Bi) and transition metals (Fe, Zn, Cr), which can adjust the fundamental properties of the nanomaterial, increase its functionality and provide wide scope for meeting the requirements of final application [51, 63, 70, 77]. Copper nanoalloys have made progress in sensing applications and enzyme-like catalytic applications. Compared to single metals, copper nanoalloys have controllable compositions, shapes, and sizes, which can affect the geometry and electronic surface structure, and thus the catalytic activity and selectivity [78, 79]. Copper nanoalloys also exhibit excellent photothermal properties, drug-carrying capacity and imaging capability, which are expected to be multifunctional therapeutic diagnostic nanoplatforms [80, 81]. Copper-incorporated nanocomposites have been considered as personalized nanoplatforms for biomedical applications due to the flexibility and versatility by cleverly combining different materials with different functional groups and structures through strategies such as in situ growth, self-assembly and epitaxial growth [82, 83].

The synthesis methods of CINMs are diversified, mainly including chemical treatment, thermal treatment, photochemical method, sonochemical method, electrochemical methods [84]. CINMs with various compositions, structures, sizes, and morphologies can be prepared by controlling experimental parameters such as reaction method, reagent, and reaction time [65]. These characteristics are closely related to the properties and applications of CINMs. Zero-dimensional copper-incorporated nanodots usually have ultra-small hydrodynamic sizes and good biocompatibility [85]. One-dimensional (1D) nanostructures, such as nanotubes, nanowires, and nanorods are widely used for sensor development due to good electrochemical catalytic properties and have also been attempted for anticancer and antibacterial applications [86, 87]. Two-dimensional copper-incorporated nanosheets (NSs) have large specific surface area and thin thickness, exhibiting high loading capacity and stimulus responsiveness, which are very attractive for stimulus-responsive therapeutic agent delivery and phototherapy [88, 89]. Three-dimensional (3D) copper-incorporated nanostructures, including hollow nanospheres, solid nanospheres, nanoflowers, nanocubes, have been more explored in biomedicine due to the superior photothermal properties and structural stability [26, 75, 90–92]. Specific functional requirements can be met by precisely adjusting the structure and morphology of CINMs. For example, hollow copper sulfide nanoparticles (HCuS NPs) are considered suitable carriers because of the tunable pore size and morphology, hollow structure, and good photothermal conversion properties. They can attach targeting ligands, imaging markers and therapeutic agents, thereby providing additional functionality and enabling the preparation of temporally and spatially controlled “smart” nanomaterials and real-time therapeutic monitoring [93, 94].

## Applications of CINMs-based PTT in Cancer therapy

**Fig. 2:**
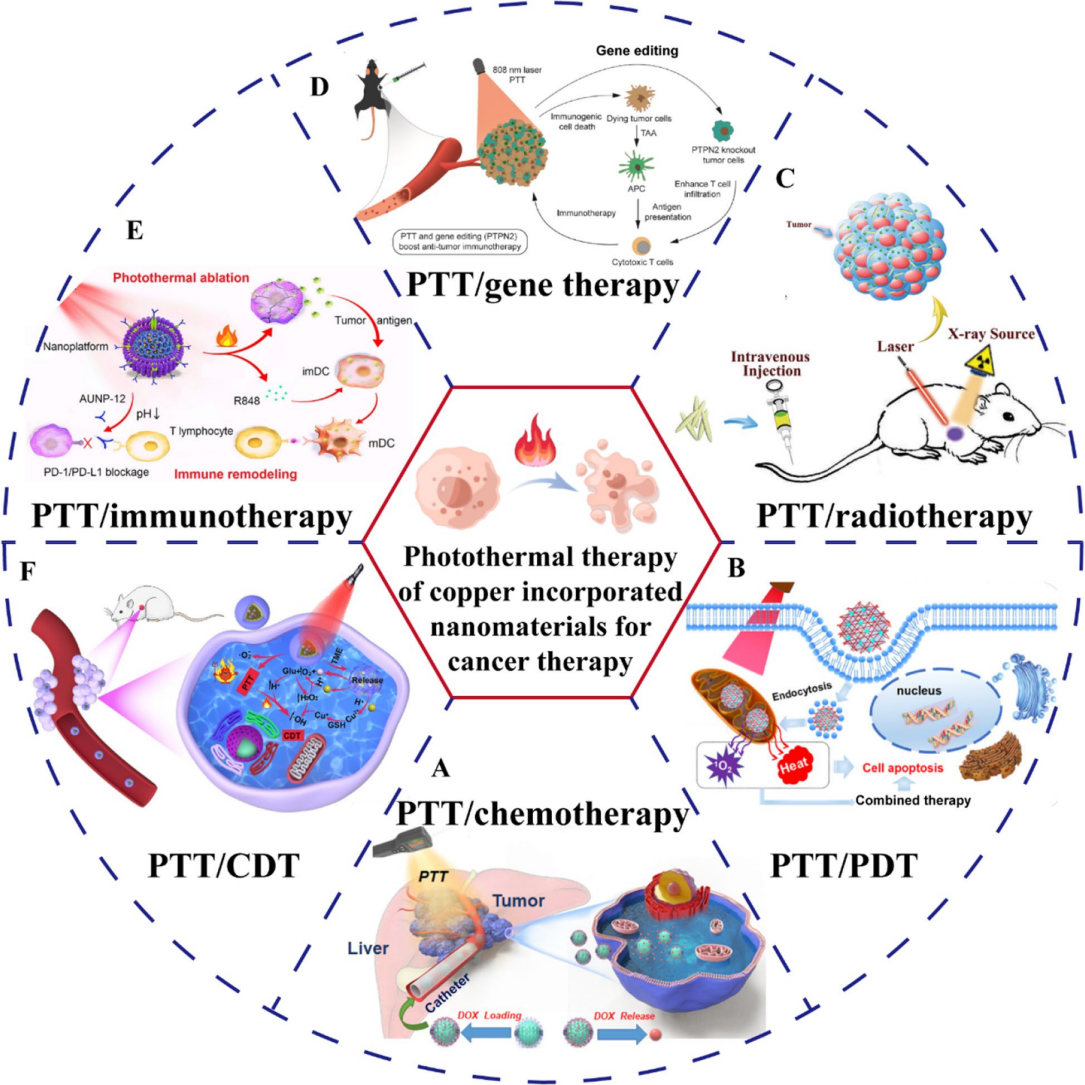
Schematics of CINMs-based PTT for cancer combination therapy. **A** Combination of PTT and chemotherapy. Reproduced with permission [99]. Copyright 2021, Elsevier. **B** Combination of PTT and PDT. Reproduced with permission [100]. Copyright 2018, American Chemical Society. **C** Combination of PTT and radiotherapy. Reproduced with permission [101]. Copyright 2020, Wiley-VCH. **D** Combination of PTT and gene therapy. Reproduced with permission [102]. Copyright 2021, Elsevier. **E** Combination of PTT and immunotherapy. Reproduced with permission [103]. Copyright 2020, American Chemical Society. **F** Combination of PTT and CDT. Reproduced with permission [67]. Copyright 2022, American Chemical Society

Compared to conventional treatments, PTT is efficient and non-invasive, however, it has various limitations [95]. For instance, the penetration depth of light limits its applications to superficial lesions. Moreover, overexpressed heat shock proteins (HSPs) can increase the ability of tumor cells to resist high temperatures, and residual tumor cells at the lesion margin often lead to tumor recurrence and distant metastasis. High-intensity lasers in therapy can damage healthy tissues around the tumor [96, 97]. Tumor heterogeneity and drug resistance can also affect the efficacy of PTT [98]. The efficacy of PTT alone is also low. Fortunately, the combination of PTT with various therapies via intelligent designs has the potential for achieving better therapeutic outcomes (Fig. 2) [104, 105].

### CINMs-based PTT/chemotherapy combination therapy

Chemotherapy is a common approach in cancer research and clinical applications. However, due to the lack of tumor specificity, the conventional chemotherapeutic drugs often lead to severe side effects [96, 106]. Multidrug resistance (MDR) may also lead to treatment failure [107]. PTT-induced increase in local temperature can facilitate chemotherapeutic drug absorption and transportation by promoting the permeability of tumor cell membranes, and also enhance the sensitivity of cancer cells to DNA-damaging drugs by disrupting DNA repair mechanisms [12]. Thus, PTT can enhance chemotherapeutic efficacy. Synergistic PTT/chemotherapy nanoplatforms with the ability to respond to the tumor microenvironment (TME) is an effective therapeutic strategy [96].

**Fig. 3:**
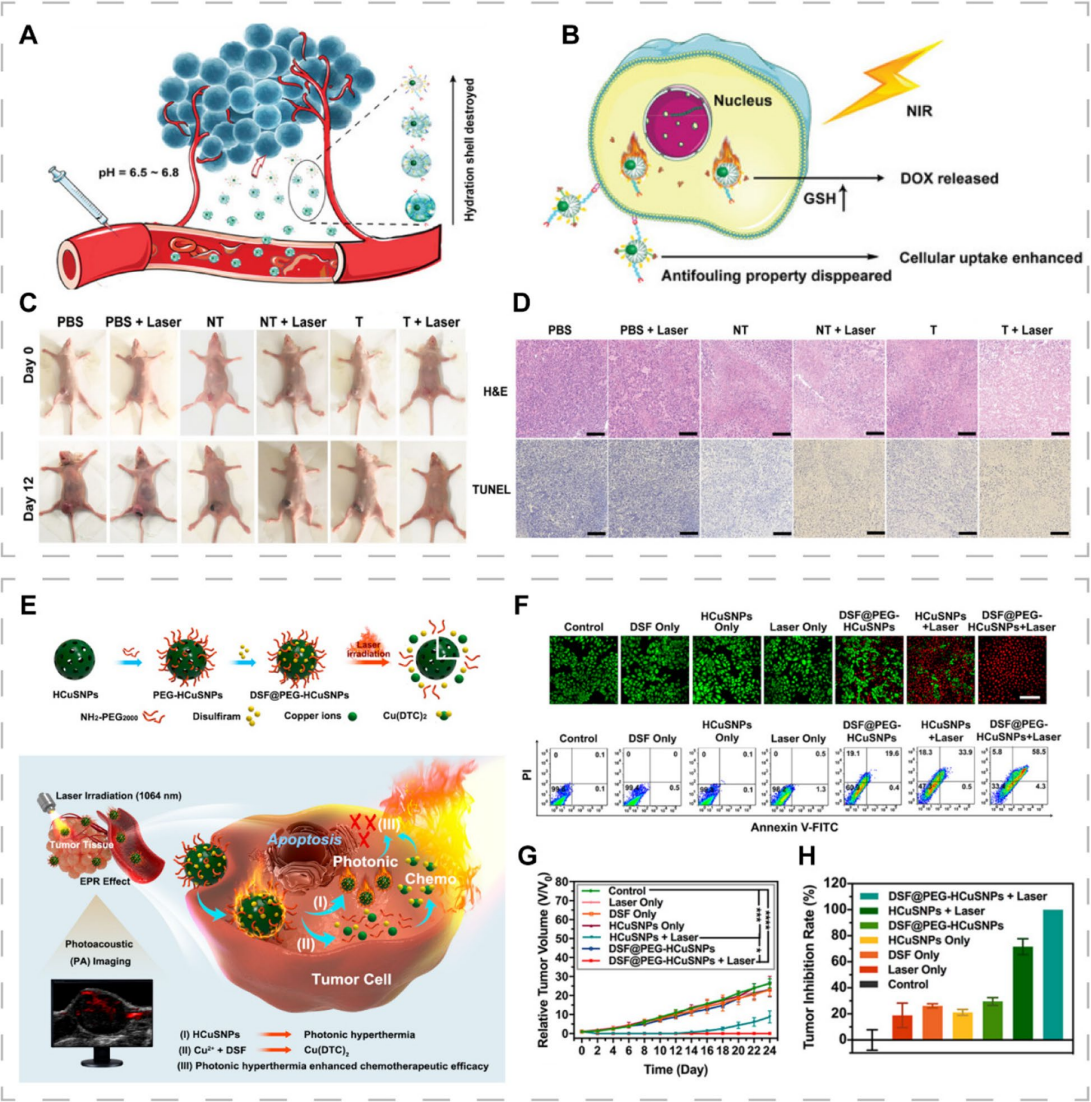
CINMs-based PTT/chemotherapy combination therapy. In vivo therapeutic mechanisms of functionalized CuS DENPs showing (**A**) anti-fouling properties and (**B**) dual responsiveness to the tumor environment. **C** Representative images of 4T1 tumor mice models after different treatments. **D** H&E and TUNEL staining of tumor tissues. Reproduced with permission [112]. Copyright 2021, Wiley-VCH. **E** Synthetic procedures and therapeutic mechanisms of DSF@PEG-HCuSNPs. **F** Live-dead staining and flow cytometry analysis after different treatments. **G** Tumor volume and (**H**) tumor inhibition rate of 4T1 tumor mice after different treatments. Reproduced with permission [113]. Copyright 2021, American Chemical Society

Chemotherapeutic drug delivery into tumors involves five steps; blood circulation, tumor accumulation, tumor penetration, cellular internalization, and drug release, in a process known as the CAPIR cascade [108]. Drug delivery systems (DDSs) have become research hotspots due to their ability to improve drug stability and biocompatibility, and to target as well as modulate drug release [109, 110]. There is a need to design DDSs that can optimally function in the aforementioned five steps, because the desired functions may play opposite roles in different steps and it is challenging to rationally coordinate them to maximize DDS functions [108, 111]. Xiong et al. developed dendrimer-entrapped copper sulfide nanoparticles (CuS DENPs) that could prolong the circulation time and increase tumor accumulation [112]. The CuS DENPs possessed dual-stimulus responsiveness of pH and redox, which were characterized by slightly acidic TME-induced charge reversal for enhanced tumor penetration/uptake and glutathione (GSH)-sensitive doxorubicin (DOX) release for improved anti-tumor effects. Copper sulfide nanoparticles (CuS NPs) enhanced chemotherapy by exerting photothermal effects in the second near-infrared (NIR-II) window (Fig. 3A, B). In vivo, CuS DENPs significantly inhibited tumor growth and had no systemic toxic effects (Fig. 3C, D). The multifunctional nanoplatform can integrate the necessary functions into one DDS, which energizes the construction of PTT combined chemotherapeutic nanoplatforms.

In vitro*,* DDSs have performed well, however, their clinical applications are limited by their unknown metabolic processing and toxicity in vivo [114]. Therefore, conversion of clinically approved drugs into anticancer drugs in specific TME is among the effective strategies. Disulfiram (DSF), a therapeutic agent for alcoholism, has been confirmed to treat cancer. In the physiological milieu, DSF can be converted to diethyldithiocarbamate (DTC), which exerts cytotoxic effects by chelating Cu^2+^ to generate Cu(DTC)_2_ [109]. This strategy requires sufficient Cu^2+^ in tumor tissues to achieve adequate antitumor effects. Liu et al. loaded DSF with polyethylene glycol (PEG)-modified HCuS NPs and constructed DSF@PEG-HCuSNPs for PTT-enhanced DSF-mediated chemotherapy (Fig. 3E–H) [113]. DSF@PEG-HCuSNPs underwent degradation in the acidic TME and released Cu^2+^ and DSF, achieving the self-supply of Cu^2+^ and in situ generation of Cu(DTC)_2_ to kill cells. This study provides a new perspective for development of novel nanoplatforms for tumor therapy by photothermal enhancement and chemical chelation reactions that enable TME-activated in situ “nontoxic to toxic” drug transformation.

Tumor cells develop drug resistance by reducing drug uptake, inactivating drugs, increasing drug efflux, and activating metabolic or detoxification pathways [115]. Clinically, MDR is one of the most important causes of chemotherapeutic failure [107]. Inhibition of overexpressed P-glycoprotein (P-gp) in tumor cells is one of the most extensively studied strategies. P-gp can promote cytotoxic drug efflux and reduce intracellular drug concentration [116, 117]. In a study of advanced hepatocellular carcinoma, Lenvatinib (LT), and Cu_2-x_S nanocrystals (NCs) were encapsulated by poly (d,l-lactide-*co*-glycolide) (PLGA). The resulting Cu_2-x_S-LT@PLGA NPs reversed the MDR properties of LT by suppressing P-gp expressions [118]. This might be because Cu_2-x_S exerted NIR-II photothermal effects to accelerate LT release, and ameliorated TME by depleting GSH and alleviating hypoxia, which suppressed P-gp. Cu_2-x_S-LT@PLGA NPs exhibited super-additive chemophotothermal therapeutic efficacy in tumor-bearing mice models, superior to monotherapy or theoretical combination therapies. Zhang et al. designed copper-palladium alloy tetrapod NPs (TNP-1) with excellent PCE and the ability to induce cytoprotective autophagy for the treatment of drug-resistant tumors [81]. They confirmed that TNP-1 induced autophagy by promoting the production of reactive oxygen species (ROS) in the mitochondria rather than by destroying lysosomes. TNP-1-mediated PTT in synergy with an autophagy inhibitor (3-methyladenine) exerted significant anti-tumor effects in drug-resistant and triple-negative breast cancer mice models. This proof-of-concept study is unique in the current context of using conventional chemotherapeutic drugs in combination with PTT against MDR.

It is undeniable that CINMs-mediated combination therapy of PTT and chemotherapy has considerable potential in anti-tumor therapy. In addition to the excellent photothermal properties, CINMs are widely used as drug carriers by virtue of the flexible nanostructures. A variety of multifunctional CINMs offer a new opportunity to solve the problem of poor effect of traditional chemotherapy. However, the current research results still have some problems that limit the clinical translation, such as the unclear therapeutic mechanism of PTT combination chemotherapy based on CINMs, the inability to predict the appropriate drug loading capacity, the fact that DDSs are usually insufficient to deliver drug doses that produce the desired efficacy, and the possibility that the encapsulated drugs may be released prematurely and lead to significant chemotherapeutic side effects [119]. To address these issues, further exploration of tumor signaling pathways and alternative mechanisms, as well as careful consideration of the genetic heterogeneity and diversity of tumors, are needed when designing CINMs for tumor synergistic PTT and chemotherapy [106]. Moreover, the rational design of enhanced passive diffusion and active targeting can be used to overcome the cellular barrier that prevents the therapeutic agent from entering the target site and ensure effective uptake by cancer cells [107].

### CINMs-based PTT/CDT combination therapy

Independent of exogenous stimuli and oxygen, CDT can generate cytotoxic hydroxyl radicals (·OH) using endogenous substances. This Fenton or Fenton-like reaction is a potential tumor treatment strategy with TME modulation and high specificity properties [120, 121]. However, conventional CDT is often limited by insufficient endogenous H_2_O_2_, the weakly acidic environment of the TME (pH 5.6–6.8), and GSH overexpression [104, 122, 123]. The copper-mediated Fenton-like reactions can occur under a wide range of pH conditions and exhibit faster reaction rates above 35 °C than the typical iron-driven Fenton reactions [124, 125]. The heat generated by PTT damages tumor cells and promotes Fenton-like reactions, thereby enhancing CDT efficacy [42, 43, 67, 126]. The PTT/CDT synergistic strategy against cancer cells has a fascinating potential for development.

**Fig. 4:**
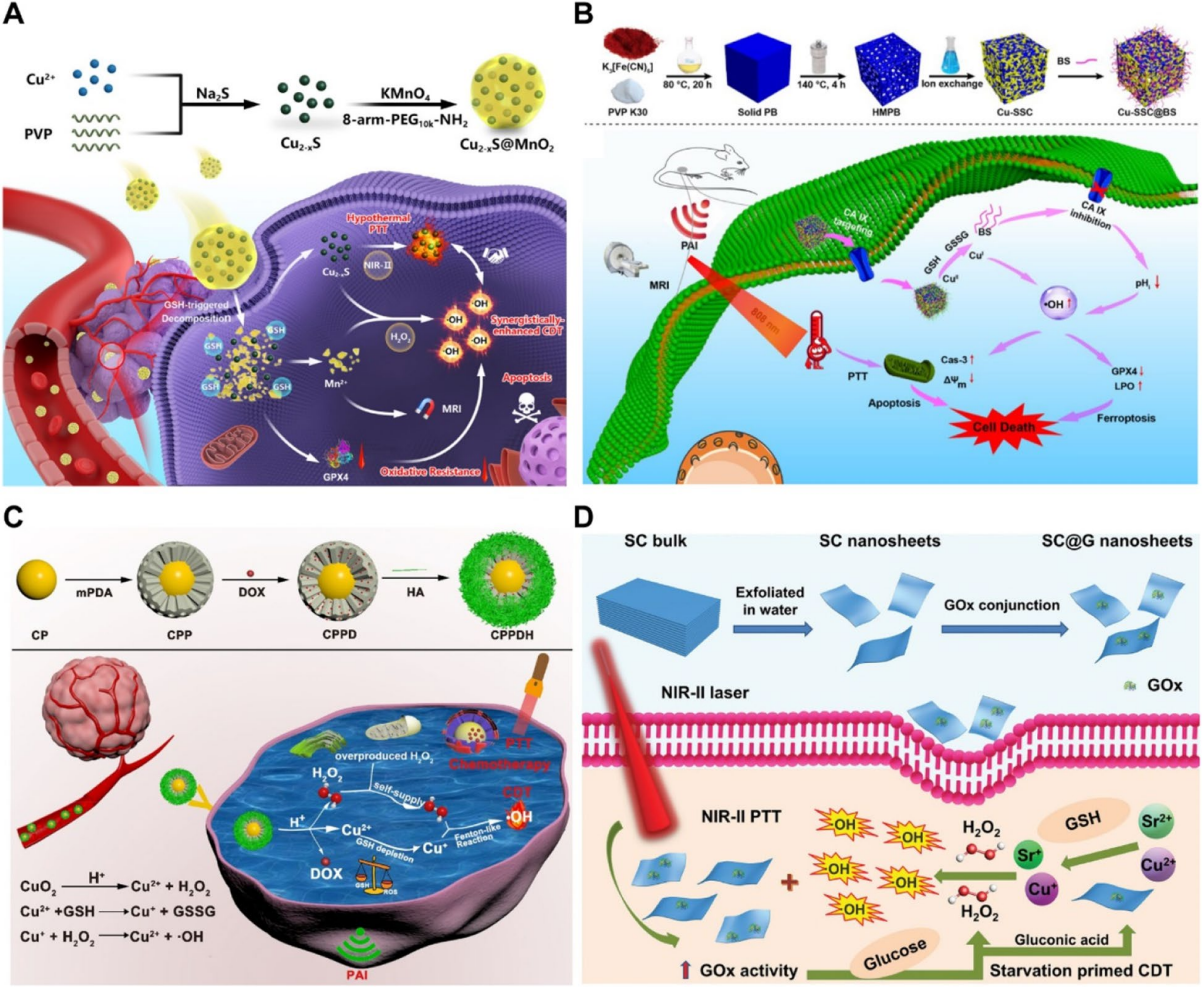
CINMs-based PTT/CDT combination therapy. **A** Cu_2-x_S@MnO_2_ nanoreactors for NIR-II hypothermal PTT and GSH consumption synergistically enhanced CDT. Reproduced with permission [128]. Copyright 2021, American Chemical Society. **B** Cu-SSC@BS for synergistic PTT and self-circulating CDT against breast cancer. Reproduced with permission [123]. Copyright 2022, Elsevier. **C** CuO_2_@mPDA/DOX-HA (CPPDH) for CDT/PTT/chemotherapy through H_2_O_2_ self-supply and GSH depletion. Reproduced with permission [105]. Copyright 2021, American Chemical Society. **D** SC@G nanosheets for synergistic NIR-II PTT-enhanced starvation/CDT against cancer. Reproduced with permission [129]. Copyright 2020, Wiley-VCH

Development of CINMs with excellent Fenton-like and photothermal properties is a major research direction. Zhang et al. developed a copper-based metal-organic framework (Cu-DBC) that significantly enhanced ·OH production by photothermally enhanced Fenton-like reaction under NIR irradiation [127]. Yao et al. prepared a nanoreactor (Cu_2-x_S@MnO_2_) capable of exerting dual-mode CDT and mild PTT (Fig. 4A) [128]. Cu_2-x_S@MnO_2_ had acidic TME-responsive copper-based catalytic properties and GSH-responsive manganese-based catalytic properties to trigger bimodal CDT. The Cu_2-x_S NPs achieved mild PTT (41.8–45 °C) due to the good photothermal properties in the NIR-II window, while the exterior manganese dioxide (MnO_2_) layer promoted oxidative stress by depleting GSH and inactivating glutathione peroxidase 4, both of which improved the catalytic performance of CDT. In vivo, Cu_2-x_S@MnO_2_ had an excellent ability to eliminate tumors in situ and inhibit distant metastases.

The highly dynamic antioxidant system in tumor cells, including antioxidant molecules and enzymes, limits the efficiency of CDT by accelerating ROS depletion [104, 130]. The TME with insufficient acidity affects the rates of Fenton-like reactions [131]. Therefore, overcoming the pH-associated limitations and increasing the oxidation potential are potential effective strategies for enhancing CDT [104]. Zuo et al. synthesized a Cu^2+^-based single-site nanocatalyst (Cu-SSC) with good photothermal performance, and combined it with 4-(2-aminoethyl) benzene sulfonamide (BS) to form a biodegradable nanocatalyst (Cu-SSC@BS) [123]. The Cu^2+^ reacted with overexpressed GSH to generate Cu^+^ for Fenton-like reactions, while disrupting the antioxidant system (Fig. 4B). The BS inhibited carbonic anhydrase IX and prevented tumor invasion as well as metastasis by suppressing extracellular matrix degradation. Moreover, BS decreased intracellular acidity to promote Cu-SSC@BS biodegradation and release of Cu^2+^ as well as BS, achieving self-cyclically enhanced CDT. The Cu-SSC@BS effectively inhibited tumor progression and metastasis under laser irradiation by synergistic PTT/self-circulating CDT.

The levels of H_2_O_2_ in tumor tissues cannot meet the demands for efficient CDT, thus, various H_2_O_2_ supply systems have emerged, which can be divided into two categories: external H_2_O_2_ delivery and endogenous H_2_O_2_ activation [16, 40, 43, 132]. Xiao et al. used copper peroxide (CuO_2_) to realize H_2_O_2_ self-supply [105]. The CuO_2_ produced Cu^2+^ and H_2_O_2_ in acidic environments, and Cu^2+^ reacted with GSH to form Cu^+^, which catalyzed H_2_O_2_ to generate ·OH (Fig. 4C). They realized the combination of PTT, CDT, and chemotherapy by loading DOX and constructing mesoporous polydopamine (PDA) on CuO_2_ surfaces, which exhibited excellent photothermal effects and could be cleaved in acidic TME.

Glucose oxidase (GOx) has been active in the field of multifunctional nanoplatforms for tumor therapy [40, 67]. The GOx can block energy supply to cancer cells by catalyzing the production of gluconic acid and H_2_O_2_ from glucose in tumors, promoting cancer cell starvation. This process achieves TME acidification and in situ H_2_O_2_ production, thereby ensuring efficient CDT [133, 134]. Based on this strategy, Yang et al. conjugated GOx with strontium copper tetrasilicate (SrCuSi_4_O_10_) to develop multifunctional SC@G NSs for synergistic NIR-II PTT-enhanced starvation/CDT against cancer [129]. The SC@G NSs exhibited high PCE (46.3%) in the NIR-II window. The generated heat enhanced the catalytic activities of GOx in tumor starvation therapy, TME acidity regulation, and H_2_O_2_ production, which enhanced CDT effects. Amplification of acidity accelerated NSs degradation and release of Sr^2+^ as well as Cu^2+^, which promoted in situ conversion of H_2_O_2_ to ·OH (Fig. 4D). This synergistic PTT/CDT/starvation therapy exhibited significant antitumor effects and good biocompatibility in 4T1 tumor-bearing mice. Intravenously administration of SC@G NSs had no significant effect on blood glucose levels.

Due to the limited efficiency of single Fenton-like reaction of CINMS, the strategy of increasing the reaction rate by PTT and designing TME-responsive CINMs has been widely recognized. Although many studies have been published, the current results still fail to achieve “zero release” of CINMs in healthy tissues [121]. Therefore, CINMs should be designed according to specific tumors to increase the enrichment in tumor tissues, thus achieving the win-win goal of maximizing efficacy and minimizing side effects. It is necessary to overcome the factors limiting the CDT reaction rate, such as insufficient acidity, insufficient H_2_O_2_, and high GSH in TME, so as to ensure the antitumor efficacy of combination therapies.

### CINMs-based PTT/PDT combination therapy

PDT is an emerging photoactivation strategy that generates cytotoxic ROS, including H_2_O_2_, ·OH, superoxide anion radicals, and singlet oxygen (^1^O_2_) by activating photosensitizers under laser irradiation at specific wavelengths [95, 135, 136]. These ROS induce cancer cell apoptosis or necrosis by directly killing cells, damaging the tumor vasculature, and activating immune responses [19, 110, 137]. Currently, PDT has been applied to skin diseases as well as certain cancers, such as esophageal and lung cancers [136, 138]. PDT requires three essential elements: photosensitizers, light, and molecular oxygen [9, 139]. The therapeutic applications of PDT are limited by its dependency on oxygen, poor tissue permeability, and uneven photosensitizer distribution [140, 141]. Mild hyperthermia of PTT can enhance cellular uptake of photosensitizers, improve tumor tissue perfusion, increase oxygen content, enhance ROS production, and promote apoptosis by destroying the mitochondria [142, 143]. Meanwhile, ROS can destroy HSPs, which play tumor cell protective roles during PTT, enhancing the efficacy of PTT [128, 144]. This PTT/PDT synergistic therapy can overcome the inadequacy of monotherapy and improve the antitumor effects of phototherapy [44, 98].

**Fig. 5:**
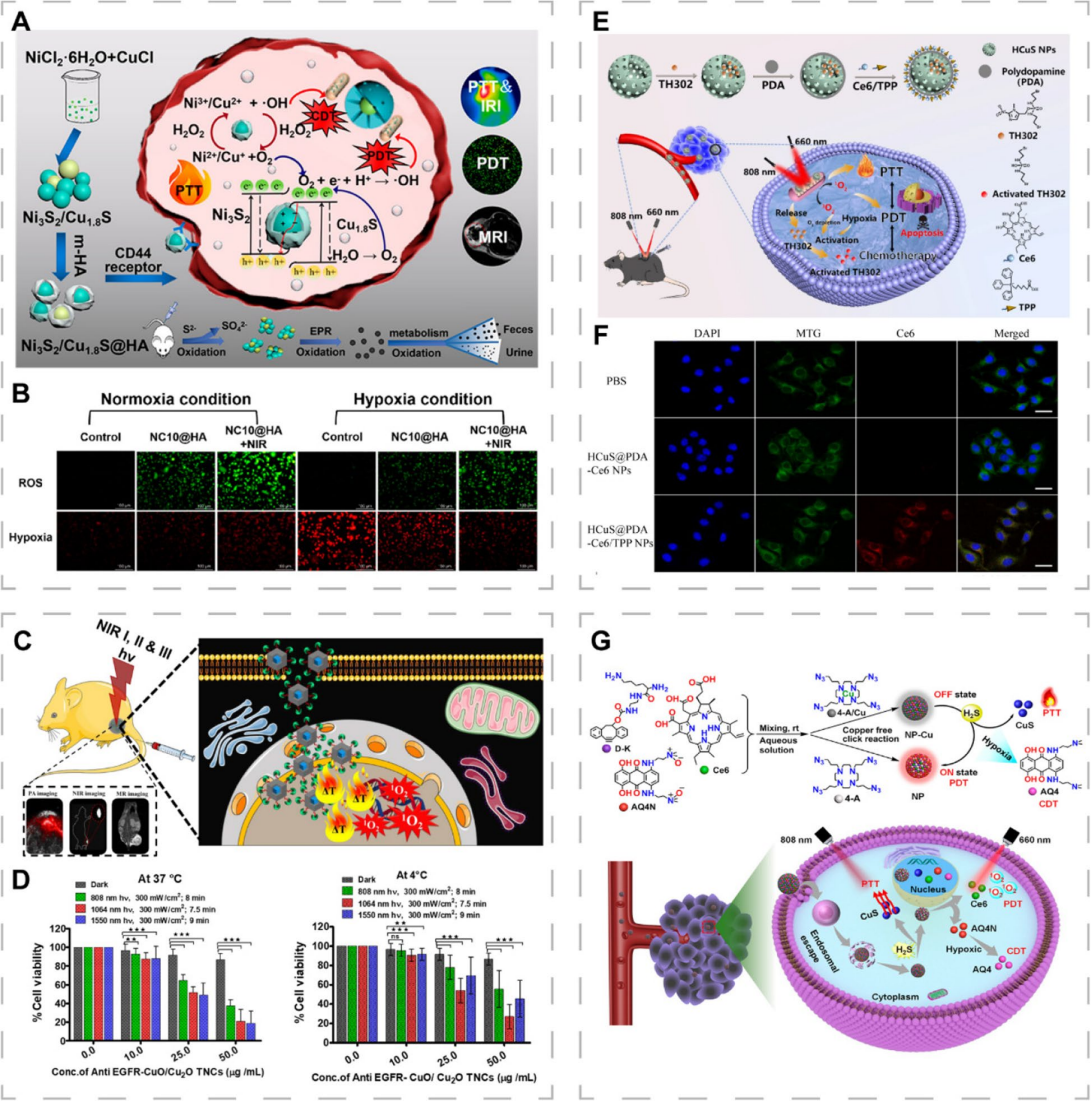
CINMs-based PTT/PDT combination therapy. **A** Synthetic procedures and therapeutic mechanisms of Ni_3_S_2_/Cu_1.8_S@HA. **B** Intracellular ROS and hypoxia levels in Mda-Mb-231 cells under normal and hypoxic conditions. NC10@HA represents Ni_3_S_2_ NPs doped with 10% Cu^2+^. Reproduced with permission [146]. Copyright 2021, American Chemical Society. **C** Schematic illustration of the in vivo experiment of CuO/Cu_2_O TNCs against drug-resistant lung cancer. **D** Viabilities of H69AR cells treated with anti-EGFR-CuO/Cu_2_O TNCs under dark and NIR light conditions at 37 and 4 °C, respectively (**p < 0.01 and ***p < 0.001). Reproduced with permission [26]. Copyright 2021, American Chemical Society. **E** Synthetic procedures and therapeutic mechanisms of HCuS-TH302@PDA-Ce6/TPP NPs. **F** Cellular uptake and mitochondrial co-localization of HCuS@PDA-Ce6 NPs and HCuS@PDA-Ce6/TPP NPs in B6F6 cells. MTG represents Mito-Tracker Green, a mitochondrial staining dye. Reproduced with permission [142]. Copyright 2022, Springer Nature. **G** Synthetic procedures and therapeutic mechanisms of NP-Cu as an endogenous H_2_S-responsive intelligent nanoplatform. Reproduced with permission [147]. Copyright 2022, American Chemical Society

Improving the hypoxic TME is essential for the efficacy of PDT [145]. Sang et al. synthesized Ni_3_S_2_/Cu_1.8_S@HA nanocomposites with high PCE (49.5%) by doping copper into nickel sulfide (Ni_3_S_2_) [146]. Ni_3_S_2_/Cu_1.8_S@HA possessed Z-scheme charge-transfer mechanisms that ensured high redox capacity and effective charge separation, which alleviated TME hypoxia by enabling intracellular photocatalytic O_2_ production and enhanced PDT. The nanocomposites had peroxidase activity that further generated more O_2_ to improve PDT (Fig. 5A, B).

The tissue penetration of NIR light restricts phototherapy to superficial tumors only, thus, there is the need to develop nanomaterials that can absorb longer light wavelengths to increase light penetration depth [9, 140]. Shanmugam et al. reported multifunctional CuO/Cu_2_O truncated nanocubes (TNCs) to treat multidrug-resistant lung tumors in deep tissues. CuO/Cu_2_O TNCs exhibited broad and extendable NIR absorption, as demonstrated by NIR-I (808 nm) /NIR-III (1550 nm) PTT as well as the combination of NIR-II (1064 nm) PDT and PTT (Fig. 5C, D) [26]. The extremely high molar extinction coefficient promoted tumor cell killing at a very low excitation light intensity (0.3 W·cm^−2^).

Ideal photosensitizers should exhibit good water solubility, stability, tumor tissue targeting ability, high quantum yield, longer wavelength absorbance, and low systemic toxicity [136]. To improve the efficacy of PDT and address the limitations of the current photosensitizers, various strategies to prevent aggregation by scaffolding uniformly dispersing photosensitizers, targeting the mitochondria, and designing activatable photosensitizers have been proposed [9, 95]. Lv et al. integrated PTT, PDT, and hypoxia-activated chemotherapy to develop a mitochondria-targeted nanoplatform (HCuS-TH302@PDA-Ce6/TPP NP) [142]. The HCuS NPs were drug carriers with good photothermal conversion properties and loaded with the thermosensitive drug (TH302) that could release the cytotoxic DNA crosslinker, bromo-isophosphoramide mustard, in the hypoxic TME. The PDA coating served as a photothermal sensitive gatekeeper to maintain HCuS NPs stability. Triphenyl phosphonium (TPP) was used to target the mitochondria, while Chlorin e6 (Ce6) acted as a photosensitizer. Therefore, NPs preferentially accumulated in the mitochondrial inner membrane to gradually activate PDT and PTT under laser irradiation at different wavelengths (660 nm and 808 nm). The generated local heat accelerated TH302 release to achieve synergistic cancer cell killing (Fig. 5E, F). This subcellular targeting strategy enhances cytotoxic activities by restricting nanomaterials to vulnerable organelles, such as lysosomes and the mitochondria, thereby preventing ROS from being consumed in the cytoplasm [39, 45, 100].

The activatable photosensitizer strategy enhances the antitumor effects of phototherapy, alleviates the potential toxicity caused by residual photosensitizers and relieves patients from the discomfort of light exposure avoidance for a long time after treatment. Yang et al. quenched the photosensitizer (Ce6) by chelating Cu^2+^ on assembled nanostructures to ensure deactivation states during cycling [147]. Cu^2+^ can react with endogenous H_2_S that is highly expressed in colon cancer to generate CuS to activate PTT in situ, while activating PDT by achieving fluorescence recovery of Ce6 (Fig. 5G). Sun et al. prepared trimodal synergistic cancer therapeutics (Cu/CDs-Ce6 NPs) by assembling Cu^2+^, Ce6, and carbon dots, which achieved a quenched state of Ce6 [148]. The functions of Ce6 were restored by overexpressing GSH and H_2_O_2_ as well as lowering the pH in the TME. This is a potential strategy for development of precision tumor therapy.

The combination of PTT and PDT is complex, and close coordination of the light absorber, light source, and therapy response monitoring should be ensured when designing a phototherapy regimen based on CINMs.Sequential treatments of PTT and PDT may be more effective than simultaneous treatments of PTT and PDT, and thus more studies are needed to select the appropriate treatment sequence and treatment interval [149]. Moreover, the development of CINMs with longer light-responsive wavelengths, high PCE, high ROS yield, and rapid degradation in vivo is a future endeavor.

### CINMs-based PTT/radiotherapy combination therapy

Radiotherapy is a classical cancer treatment approach that induces apoptosis and necrosis in a non-invasive manner. It is usually classified as external beam radiotherapy, radionuclide therapy, and brachytherapy [150]. Radiotherapy utilizes high-energy ionizing radiation to damage the DNA directly or indirectly by reacting with water molecules to produce ROS [151]. However, hypoxia in the TME limits the effects of radiotherapy and mediates tumor cell resistance to radiotherapy, as hypoxia-induced malignant clones, immune evasion, and interference with DNA damage responses promote cancer progression [141, 152, 153]. Mechanistically, PTT can enhance hypoxic cancer cell sensitivity to radiotherapy by increasing perfusion and improving oxygenation [12, 154].

Many nanoplatforms incorporating PTT and radiotherapy can promote oxygen levels in the TME to achieve oxygen self-replenishment, thereby enhancing the efficacy of radiotherapy [151, 155]. Jiang et al. synthesized an oxygen self-supply system (CuS@CeO_2_ NPs) consisting of ultrafine CuS NPs and mixed valenced Ce element [101]. The nanoenzyme CeO_2_ catalyzed oxygen generation from endogenous H_2_O_2_ in tumor cells. The ultrafine CuS NPs were released deep into tumor cells and played dual roles of secondary radical emitters and PTT agents under X-ray and NIR-II irradiation, respectively. The spindle-shaped CuS@CeO_2_ NPs were more conducive to cellular endocytosis and could synergize with radiotherapy as well as PTT to treat hypoxic tumors. This study achieved long-term alleviation of hypoxia by remodeling the TME into a tumor niche that is conducive to radiotherapy by catabolizing endogenous H_2_O_2_ and photothermally increasing perfusion, which provides a positive strategy for radiosensitization of deeply hypoxic tumors.

**Fig. 6:**
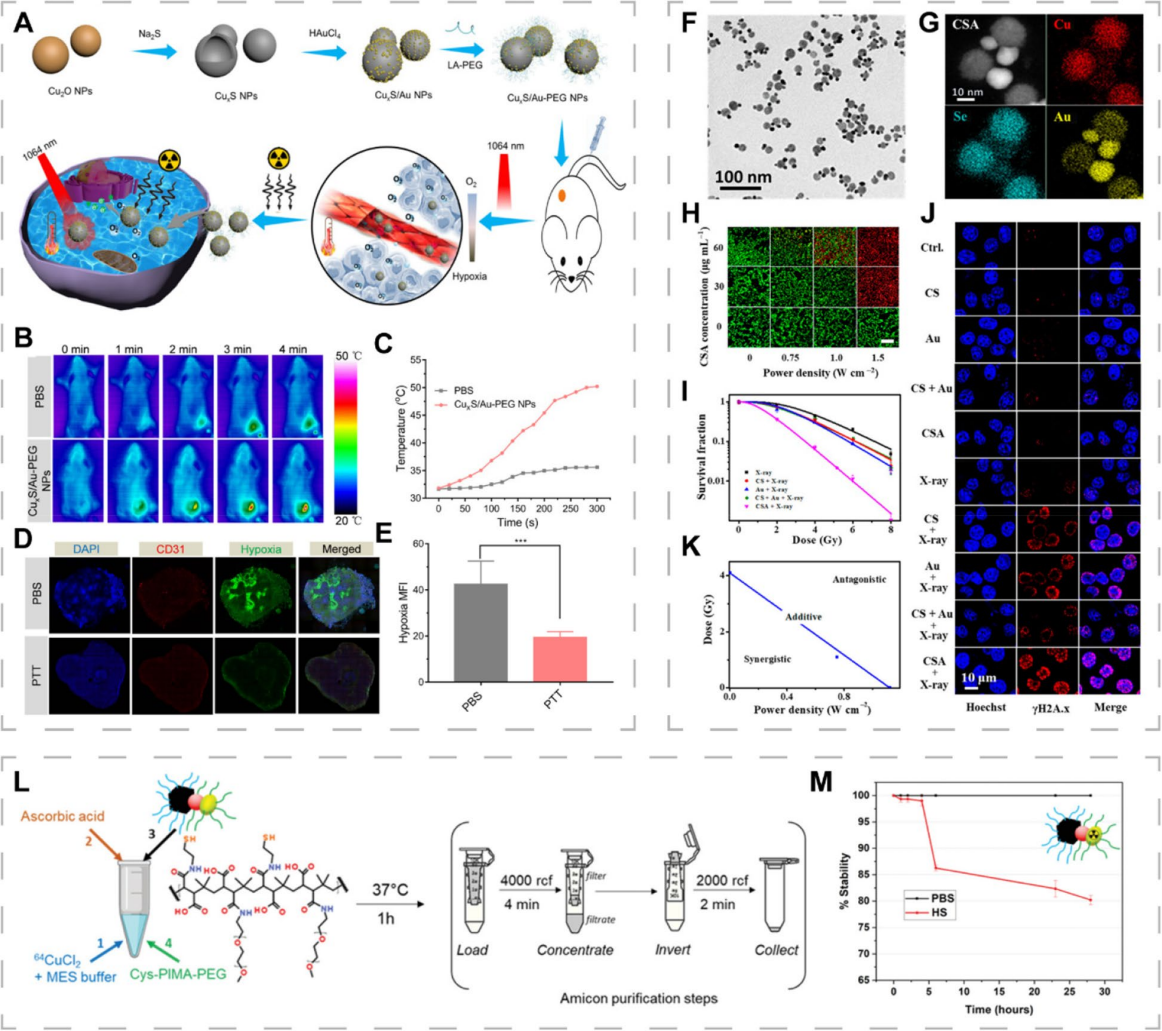
CINMs-based PTT/radiotherapy combination therapy. **A** Synthetic procedures and therapeutic mechanisms of Cu_x_S/Au-PEG NPs. **B** Thermal images and **C** photothermal conversion abilities of Cu_x_S/Au-PEG NPs under 1064 nm laser irradiation in tumor-bearing mice. **D** Immunofluorescence images and (**E**) quantification of tumor hypoxic regions after NIR irradiation (***p < 0.001). Reproduced with permission [158]. Copyright 2020, American Chemical Society. **F** Transmission electron microscopy (TEM) image and (**G)** high-angle annular dark-field-scanning TEM energy dispersive spectrometer elemental mapping image of CSA NPs. **H** Live-dead staining of 4T1 cells treated with different power densities of NIR light and different CSA NPs concentrations. **I** Cell survival rates and (**J)** DNA damage images of 4T1 cells after different treatments. **K** Isobologram analysis of synergistic inhibition of 4T1 cells treated with CSA NPs by applying laser and X-rays. Reproduced with permission [73]. Copyright 2019, American Chemical Society. **L** Schematic presentation of the scheme for radiolabeling and purification of Fe_3_O_4_@Au@Cu_2-x_S using ^64^Cu. **M** In vitro stability of ^64^Cu: Fe_3_O_4_@Au@Cu_2-x_S in PBS and human serum at 37 °C. Reproduced with permission [159]. Copyright 2022, Wiley-VCH

Due to the relatively small differences in responses of normal and tumor tissues to ionizing radiation, repeated high doses of ionizing radiation may damage healthy tissues around the tumor to cause serious toxic side effects to patients [96]. Various high effective atomic number (*Z*) nanomaterials (gold, bismuth, wolfram, gadolinium, and platinum) have been developed as radiosensitizers to facilitate radiation energy deposition into tumors, thereby reducing repeated exposures to high radiation doses [141, 156, 157]. Zhang et al. synthesized Cu_x_S/Au NPs (1 < x < 2) that can be used for thermal radiotherapy in the NIR-II window (Fig. 6A) [158]. Integration of gold and Cu_x_S altered the electron transitions of Cu_x_S, resulting in high PCE (44.2%) of Cu_x_S/Au NPs (Fig. 6B, C). Tumor cell oxygenation was improved under 1064 nm laser irradiation (Fig. 6D, E). Huang et al. reported dumbbell-shaped heterogeneous copper selenide-gold (Cu_2-x_Se@Au, CSA) NPs as radiosensitizers for synergistic photothermal radiotherapy (Fig. 6F, G) [73]. The CSA heterostructures exhibited higher *Z* and more severe DNA damage in tumor cells compared to the fragments alone and their mixtures (Fig. 6I, J). The CSA heterostructures also exhibited enhanced PCE (80.8%) and had a great potential for PTT (Fig. 6H). Isogram analysis showed a synergistic antitumor effect of PTT and radiotherappy after CSA heterostructures treatment (Fig. 6K). Li et al. synthesized Cu_3_BiS_3_ nanorods that induced PTT under NIR-II irradiation and deposited radiation energy. Therefore, they can be used to achieve synergistic thermoradiotherapy [41]. Nanomaterials with both radiosensitization and photothermal effects are becoming a research hotspot.

CuS, which is achieved by replacing copper atoms with radioisotope ^64^Cu or by directly adding radiolabels such as ^131^I or ^64^Cu, is one of the most commonly applied NPs for radionuclide therapy [150]. Fiorito et al. constructed a heterostructure (Fe_3_O_4_@Au@Cu_2-x_S) that was the first multifunctional nanoplatform to integrate PTT, radiotherapy and magnetic hyperthermia (MHT) [159]. Gold and Cu_2-x_S acted as PTAs to excite PTT while Fe_3_O_4_ excited MHT, forming a dual heating platform that is based on PTT and MHT. The stable insertion of ^64^Cu provided the possibility of Fe_3_O_4_@Au@Cu_2-x_S as an internal radiotherapeutic agent (Fig. 6L, M). This was a proof-of-concept study and has not been validated by cellular or in vivo experiments. Liu et al. prepared ^131^I-labeled HCuS NPs loaded with paclitaxel to achieve synergistic PTT/radiotherapy/chemotherapy for orthotopic breast cancer [160]. They also used microspheres for hepatic artery embolization to treat hepatic tumors [94]. Microspheres can improve local therapeutic effects at relatively low doses, while imaging guidance allows precise control of treatment with minimal damage to surrounding healthy tissues and other organs.

PTT synergized with radiotherapy is a promising strategy for radiosensitization. It is necessary to develop CINMs with both radiosensitizing and photothermal properties and to add imaging capabilities to provide accurate positional structural information to guide precise radiotherapy. Standardized processes need to be established for the design and use of CINMs, such as determining the sequence of NIR laser and X-rays [96]. The mechanisms of radiosensitization also need to be explored in depth. Undeniably, PTT/radiotherapy is an area full of therapeutic potential for continued development and exploration.

### CINMs-based PTT/immunotherapy combination therapy

Even though PTT can suppress primary tumors, it cannot treat recurring and distant metastatic tumors, which are associated with poor prognostic outcomes for cancer patients [161]. Immunotherapeutic approaches for activating the host’s own natural defenses to recognize and attack aggressive tumor cells have attracted attention [46, 47]. However, most cancers are immune tolerant. The PTT can induce cancer cell conversion from non-immunogenic to immunogenic, along with release of damage associated molecular patterns and tumor-associated antigens (TAAs). This process, referred to as immunogenic cell death (ICD), can activate strong immune responses [162, 163]. CINMs can also induce tumor-associated macrophages to produce ROS via Fenton-like reactions, promote macrophage polarization towards the M1 phenotype and remodel the tumor immunosuppressive microenvironment (TIM) [75]. Photo-immunotherapy (PIT) combines PTT and immunotherapy to maximize their respective advantages and improve anti-tumor efficacy [74, 164].

**Fig. 7:**
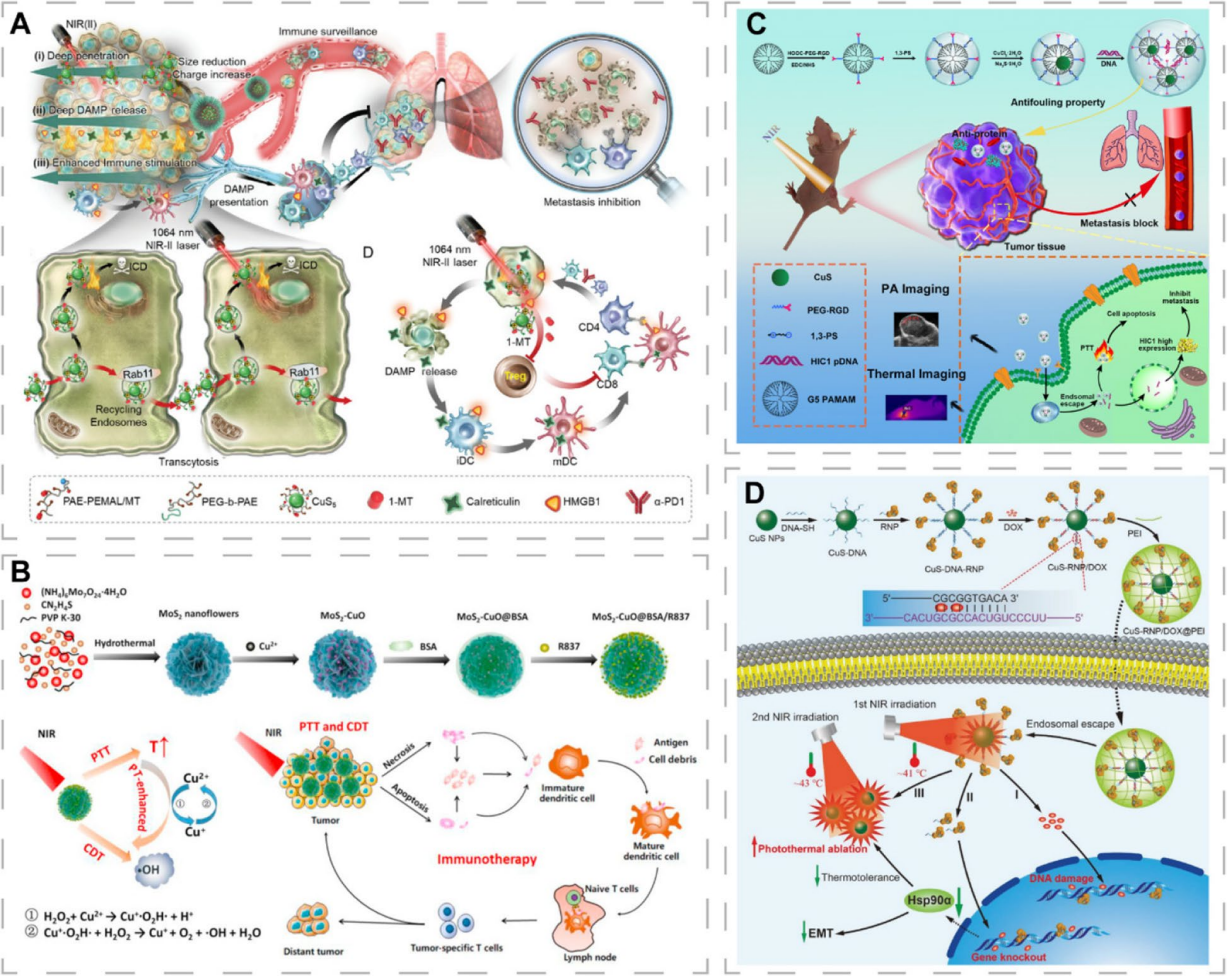
CINMs-based PTT/immunotherapy and CINMs-based PTT/gene therapy combination therapy. **A** Schematic of the mechanism of PRN^MT^-mediated PTT/immunotherapy. Reproduced with permission [74]. Copyright 2022, Wiley-VCH. **B** Synthetic procedures and therapeutic mechanisms of MoS_2_-CuO@BSA/R837 for synergistic PTT/immunotherapy/CDT. Reproduced with permission [66]. Copyright 2021, Elsevier. **C** Synthetic procedures and therapeutic mechanisms of RGD-CuS DENPs. Reproduced with permission [28]. Copyright 2021, American Chemical Society. **D** Synthetic procedures and therapeutic mechanisms of CuS-RNP/DOX@PEI for NIR-triggered Cas9 RNP and DOX delivery. Reproduced with permission [167]. Copyright 2021, Wiley-VCH

The low abundance of tumor-infiltrating lymphocytes within tumors and TIM can lead to tumor resistance to immunotherapy. Immune checkpoint blockade (ICB) strategies are widely used to reverse TIM by blocking immunosuppressive pathways and activating the immune system with immune checkpoint inhibitors. In recent years, programmed cell death protein 1 and its ligands (PD-1/PD-L1), indoleamine-2,3 dioxygenase (IDO), as well as cytotoxic T-lymphocyte-associated protein 4 have been the most investigated immune checkpoint molecules [165, 166]. The overexpression of IDO-1 in tumor cells can mediate TIM by affecting cytotoxic T lymphocytes (CTLs) and immunosuppressive regulatory T cells (Tregs). Li et al. prepared a programmed raspberry-structured nanoplatform (PRN^MT^) consisting of small-sized CuS NPs (CuS_5_) and the IDO inhibitor (D)-1-methyltryptophan prodrug (1-MT) for NIR-II PIT of deep tumors [74]. The neutrally charged PRN^MT^ could split into surface cationized CuS_5_ in acidic TME, which rapidly penetrated deep into the tumor. Under NIR-II irradiation, CuS_5_ exhibited excellent photothermal properties, induced ICD, and released 1-MT, which alleviated IDO-1-induced immunometabolic disturbances, increased the effective infiltration of CTLs, and down-regulated Tregs (Fig. 7A). In vivo, the combination of PRN^MT^ and PD-1 blockade effectively inhibited primary breast cancer growth and lung metastasis in mice.

The immune response generated by PTT-induced ICD alone may be transient and weak, and not effective enough to halt tumor progression [168]. Immune adjuvants, such as cytosine-phosphate-guanine (CpG) oligodeoxynucleotides (ODNs), ovalbumin, and R837 can be used as nonspecific immunopotentiators to enhance tumor antigen immunogenicity [47, 48, 168]. Jiang et al. loaded bovine serum albumin (BSA) and R837 on surfaces of molybdenum disulfide-copper oxide (MoS_2_-CuO) heteronanocomposites to obtain MoS_2_-CuO@BSA/R837 (MCBR) nanoplatforms, which can achieve synergistic tumor therapy with PTT/CDT/immunotherapy [66]. Tumor cells were destroyed by PTT and photothermal-enhanced CDT, and the released TAAs bound R837 to activate the immune system by promoting dendritic cell (DC) maturation, secreting cytokines, and increasing lymphocyte counts, thereby inhibiting primary and metastatic tumor progression (Fig. 7B). The combination of PTT with immune adjuvants and ICB therapy can result in a “doomsday storm” to tumors by enhancing immunogenicity and activating the immune system. Cheng et al. prepared a smart biomimetic nanoplatform (AM@DLMSN@CuS/R848) by loading the immune adjuvant resiquimod (R848), and the PD-1/PD-L1 peptide inhibitor AUNP-12 [103]. AM@DLMSN@CuS/R848 exhibited vaccine-like functions and enhanced T lymphocyte functions, allowing PTT and immune remodeling to synergistically act to enhance the treatment of metastatic triple-negative breast cancer.

The mechanism of CINMs-based PIT has not been fully elucidated. Given the dynamic and complex nature of the immune system, where PIT can induce unpredictable changes through amplified circuits and attack the immune system itself, there is a need to explore the optimal temperatures that can stimulate the maximum killing power of PTT and regulate immune system responses. In addition, avoiding the formation of metastatic tumors by killing circulating tumor cells in the bloodstream through PIT is a future challenge to be tackled.

### CINMs-based PTT/gene therapy combination therapy

Gene therapy has been shown to improve the efficacies of antitumor therapies by restoring the expressions of dysregulated genes to kill cancer cells without harming the normal tissues. However, clinical applications of gene therapy are limited by poor stabilities of delivery systems, low transfection efficiencies and non-specific effects [96, 107].

The combination of PTT and gene therapy can produce synergistic therapeutic effects and enhance gene delivery. This is because PTT can enhance cellular uptake, endosomal escape after internalization and gene release [169]. Ouyang et al. performed a study to simultaneously inhibit primary and metastatic tumors (Fig. 7C) [28]. They successfully developed a tumor-targeted therapeutic platform combining PTT and gene therapy by integrating CuS DENPs, arginine-glycine-aspartate (RGD) peptides, and plasmid DNA-encoding hypermethylation in cancer 1 (pDNA-HIC1). The platform achieved a PCE of 49.8% and enhanced serum delivery of pDNA-HIC1 to inhibit cancer cell invasion as well as metastasis.

Clustered regularly interspaced short palindromic repeats-associated protein 9 (CRISPR-Cas9) technology has an extraordinary potential for gene editing. On-demand delivery and activation of CRISPR-Cas9 via photothermal effects to improve efficacy and reduce side effects is a research hotspot [170]. Chen et al. developed an NIR-triggered nanotherapeutic platform (CuS-RNP/DOX@PEI) and achieved controlled drug release as well as gene editing by synergistic PTT/gene therapy/chemotherapy (Fig. 7D) [167]. They coupled Cas9 ribonucleoprotein (RNP) and CuS NPs with thiol-modified DNA fragments, followed by DOX insertion and coated with endocytosis-promoting polyethylenimine (PEI). The release of Cas9 RNP and DOX was achieved by double-chain breaks mediated by photothermal effects of CuS (≈41 °C). Cas9 RNP depleted Hsp90α, which promoted tumor invasion as well as metastasis, thereby suppressing tumor tolerance to heat and inhibiting tumor metastasis. CuS-RNP/DOX@PEI with NIR irradiation in xenograft BALB/c mice loaded with A375 tumor achieved photothermal control of gene editing and exhibited significant tumor suppressive effects. Tao et al. used CuS-RNP@PEI NPs to deliver the Cas9 RNP targeting protein tyrosine phosphatase non-receptor type 2 (PTPN2). Depletion of PTPN2 improved antigen presentation as well as CD8 T lymphocyte accumulation, enabling photothermal responsive gene editing in combination with immunotherapy [102].

Compared to other synergistic therapies, photothermal combined gene therapy based on CINMs has been less studied and clinical translation has not been attempted. The efficacy of gene therapy depends on the efficiency of the gene delivery system to deliver the intact exogenous gene to the nucleus. However, instability and suboptimal bioavailability of gene carriers can affect the efficacy. The design of photothermally responsive nucleus-targeted CINMs may be effective. The biocompatibility of CINMs-based gene delivery systems is also a critical issue and more efforts need to be invested to overcome these challenges [169].

## CINMs for image-guided PTT in cancers

With rapid nanotechnological advances, development of nanoplatforms that enable tumor tissue visualization has become an important goal [114]. They can guide treatment by reporting information on 3D spatial location, structures of malignant tumors and monitoring treatment progress in real time, enabling intelligent imaging as well as precise treatment [98, 171, 172]. The potential applications of CINMs as contrast agents for PAI, fluorescence imaging, computed tomography (CT) imaging, magnetic resonance imaging (MRI), positron emission tomography (PET) imaging, and single photon emission computed tomography (SPECT) imaging in tumor imaging has been extensively investigated [50–53, 173]. Multimodal imaging revolutionizes the field by seamlessly integrating multiple imaging techniques onto a single nanoplatform. This cutting-edge approach capitalizes on the unique strengths of each method, thereby enhancing sensitivity, spatial resolution, and the wealth of information available for guiding treatment decisions. Designing multifunctional nanomaterials for multimodal imaging-guided PTT has become the current research trend [97, 171].

### CINMs for MRI-guided PTT

**Fig. 8:**
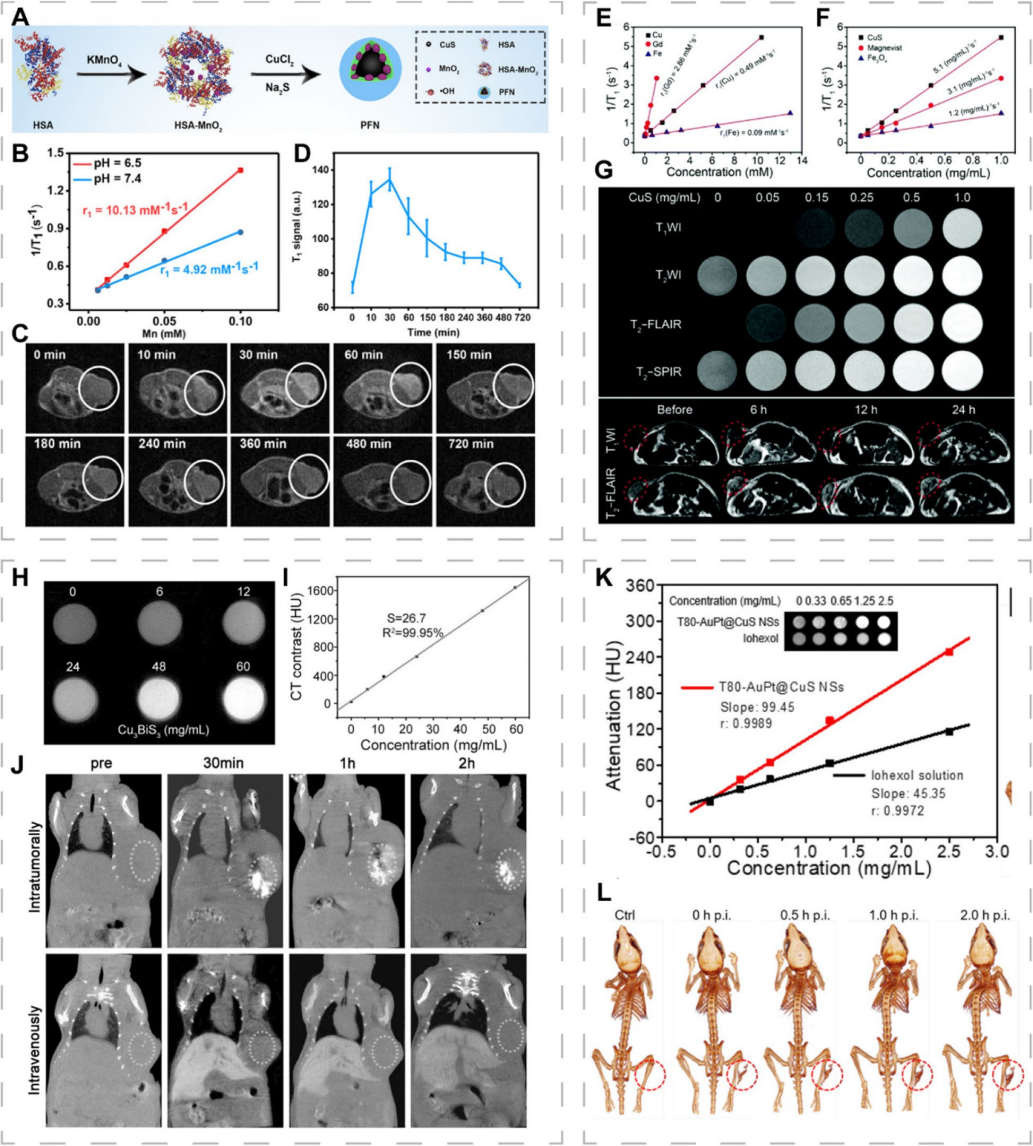
CINMs for MRI- and CT-guided PTT. **A** Synthetic procedures of PFN. **B** Longitudinal relaxation rate (*r*_1_) of PFN. **C** T_1_WI and (**D**) signal intensities of tumor-bearing mice at various time intervals after PFN treatment. Reproduced with permission [177]. Copyright 2020, American Chemical Society. Linear fits of 1/T_1_ for CuS HNs, Magnevist (T_1_WI contrast agent) and Fe_3_O_4_ (T_2_WI contrast agent) at different (**E**) molar concentrations of Cu, Gd and Fe and (**F**) mass concentrations. **G** In vitro MRIs of different mass concentrations of CuS + DOX aqueous dispersions, and MRI of tumor-bearing mice treated with CuS + DOX (1.0 mg mL^−1^, 100 μL). Reproduced with permission [90]. Copyright 2018, Royal Society of Chemistry. **H** In vitro CT images of different concentrations of Cu_3_BiS_3_ NCs aqueous dispersions. **I** CT value as a function of Cu_3_BiS_3_ NCs concentration. **J** CT coronal images of tumor-bearing mice after intratumoral injection and intravenous injection of Cu_3_BiS_3_ NCs. Reproduced with permission [77]. Copyright 2015, Wiley-VCH. (K) Linear fit of HU values of T80-AuPt@CuS NSs and iohexol solution at different concentrations. **L** CT images of tumor-bearing mice treated with T80-AuPt@CuS NSs. Reproduced with permission [156]. Copyright 2021, American Chemical Society

Due to its excellent spatial resolution and good depth of tissue penetration, MRI is widely used for molecular and cellular imaging [174, 175]. Incorporation of metal ions containing a large number of unpaired electrons is a feasible strategy for endowing PTA with MRI functions [176]. Qi et al. designed Tpc-CuGd NPs composed of copper and gadolinium and modified by transferrin protein corona to enable MRI-guided tumor-targeted photothermal and chemodynamic therapy [126]. The Tpc-CuGd NPs with a Cu/Gd ratio of 4/4 exhibited the best PCE (55.8%) and catalytic activities. Addition of Gd ions significantly enhanced the T_1_-weighted imaging (T_1_WI) signal without obvious toxicity. Mn^2+^ exhibited excellent paramagnetic properties and can be used as a T_1_WI contrast agent. Sun et al. reported a photothermal Fenton nanocatalyst (PFN) composed of CuS, human serum albumin and MnO_2_, in which CuS served as a PTA and Fenton catalyst (Fig. 8A) [177]. PFN could decompose Mn^2+^ in the acidic TME to increase relaxivity by 2.1-fold and enhance T_1_WI signal intensity, achieving synergistic PTT and CDT of pancreatic cancer under MRI guidance (Fig. 8B-D).

Given the complexity, difficulty and potential side effects of synthesizing multi-component composite nanomaterials, it is better to develop single-component nanomaterials with photothermal and imaging abilities [77, 172]. Cu^2+^ has an unpaired 3d electron and is a potential MRI contrast agent [178]. However, Cu^2+^ presents poor contrast because the outermost orbital only has one unpaired electron. To improve imaging performance, based on the theory that enhanced direct contact between metal ions and water molecules can accelerate spin-lattice relaxation, Zhang et al. synthesized 3D CuS hollow nanoflowers (CuS HNs) with the ability to expose more cupric centers [90]. The loading capacity and good PCE (30%) of CuS HNs conferred chemotherapeutic and PTT potential. In vitro, CuS HNs performed well on T_1_WI and T_2_-weighted fluid-attenuated inversion recovery imaging (T_2_-FLAIR) sequences. The subsequent in vivo assays confirmed that CuS HNs could be used for T_2_-FLAIR MRI-guided PTT/chemotherapy synergistic treatment (Fig. 8E-G). Synthesizing multifunctional nanoplatforms in a simple and green way is a major trend, and more attention should be paid to the imaging potential of single-component CINMs.

### CINMs for CT-guided PTT

CT is a non-invasive imaging technique that presents high-resolution anatomical structures by generating cross-sectional images depending on X-ray attenuation degree in different tissues [179, 180]. The CT contrast agents can help distinguish among tissues with the same attenuation degree and enhance the imaging effects. Considering the limited X-ray absorption capacity of the currently used contrast agents, there is a need to develop nanomaterials with high X-ray attenuation coefficients as contrast agents [181, 182]. Cai et al. prepared T80-AuPt@CuS NSs using AuPt NPs coating and Tween 80 functionalization, integrating CT and synergistic photothermal radiotherapy [156]. The T80-AuPt@CuS NSs exhibited high X-ray attenuation abilities to enhance CT signals, thus, it is a promising contrast agent for CT (Fig. 8K, L).

Introducing heavy metals with high X-ray attenuation coefficients is a potential strategy for preparation of multifunctional nanoplatforms with integrated CT and PTT. Studies have reported on the potential applications of this strategy, such as introduction of BiOI, GeO_2_, and CuWO_4_ NPs (Fig. 8H–J) [183–185]. To improve CT sensitivity, coupling targeting elements can help the contrast agent accumulate in the tissue of interest. With advances in CT imaging technologies, researchers have focused on X-ray attenuation abilities as well as phase shift or scattering. In future, more NPs with X-ray scattering properties will be used for CT-guided PTT [186].

### CINMs for PET/SPECT-guided PTT

The PET and SPECT are two emerging non-invasive nuclear imaging techniques that have the ability for visualizing metabolic processes at cellular and molecular levels. PET uses positron-emitting radionuclide labeled imaging agents, whereas SPECT uses radionuclides that directly emit γ-rays [187]. They exhibit excellent sensitivity and quantification, but perform poorly in terms of anatomical resolution [188]. They have attracted attention in evaluation of tumor uptake and pharmacokinetics of nanomaterials [21, 54].

**Fig. 9:**
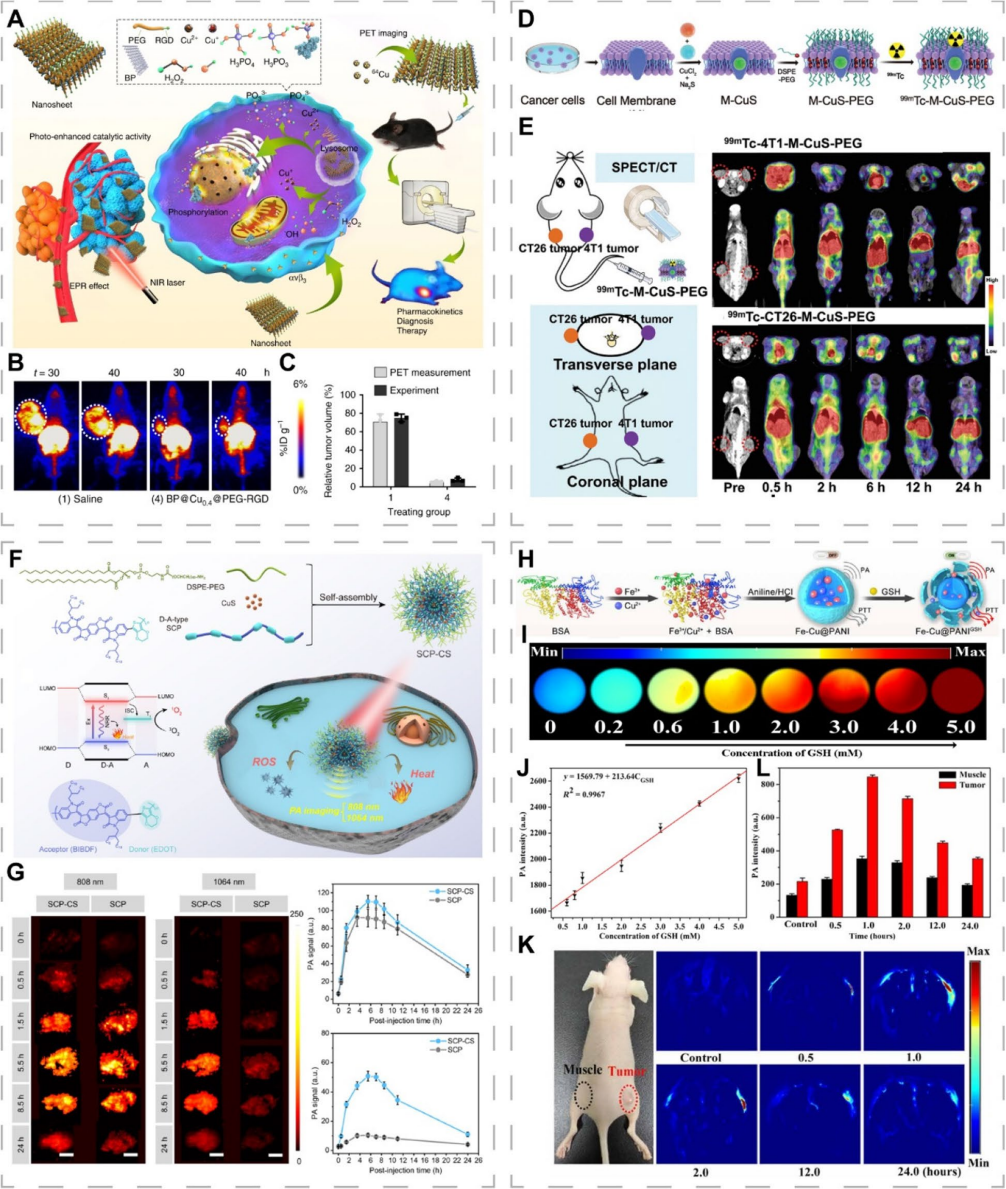
CINMs for PET-, SPECT- and PAI-guided PTT. **A** Synthetic procedures and mechanisms of BP@Cu@PEG-RGD NSs. **B** PET images of tumor-bearing mice treated with saline or NSs after 2 weeks. **C** Comparison of PET images with experimentally measured tumor volumes. Reproduced with permission [190]. Copyright 2020, Springer Nature. **D** Synthetic procedures of ^99m^Tc-M-CuS-PEG. **E** SPECT/CT images of tumor-bearing mice treated with ^99m^Tc-4T1-M-CuS-PEG or ^99m^Tc-CT26-M-CuS-PEG. Reproduced with permission [191]. Copyright 2021, Wiley-VCH. **F** Synthetic procedures and mechanisms of SCP-CS. **G** PAIs and corresponding signal intensity of tumor-bearing mice treated with SCP-CS and SCP at 808 nm and 1064 nm. Reproduced with permission [192]. Copyright 2021, Elsevier. **H** Synthetic procedures and activation mechanisms of Fe-Cu@PANI. **I** PAIs with different GSH concentrations. **J** Linear relationship between PA intensity and GSH concentration. **K** PAIs and (**L**) corresponding signal intensity of tumor-bearing mice treated with Fe-Cu@PANI. Reproduced with permission [193]. Copyright 2021, American Association for the Advancement of Science (AAAS)

In vivo stability of radionuclide-labeled nanoplatforms is critical, as separation of radionuclides from NPs can lead to a high uptake of non-target tissues, thereby reducing the reliability of imaging results and may affect subsequent treatment decisions [189]. The utilization of tight junctions between radiolabeled imaging agents and non-radioactive components in PET imaging holds tremendous promise as a potential solution to this problem. Hu et al. exploited this feature to label BP@Cu@PEG-RGD NSs with ^64^Cu^2+^ via the “chelator-free” method [190]. The whole process took 10 min to achieve 99% labeling rate and the labeled NSs exhibited good stability. The combination of Cu^2+^ with black phosphorus NSs (BPNSs) enhanced the photothermal stability and accelerated the degradation, ensuring excellent PCE while avoiding potential safety issues. The RGD-conjugated PEG coating improved the targeting ability as well as biocompatibility, and increased nanomaterial uptake and accumulation in tumor tissues, enabling quantitative and accurate tracking of biodistribution (Fig. 9A-C).

The wide applications of SPECT imaging are associated with its low equipment costs and longer half-life radioisotope [194]. Yi et al. prepared a tumor-targeted photothermal therapeutic agent (^99m^Tc-M-CuS-PEG) for SPECT imaging guidance by labeling cancer cell membrane-encapsulated CuS NPs with ^99m^Tc [191]. They found that radionuclides ^99m^Tc induced G2/M cell cycle arrest and overexpressions of endocytosis-associated protein (caveolin-1) to enhance NP uptake by tumor cells, thereby improving the photothermal efficiency in the NIR-II window. ^99m^Tc-M-CuS-PEG exhibited good radiolabeling stability, homologous tumor targeting ability, biosafety, and enhanced PCE (Fig. 9D, E). Whichever radioisotope is chosen to track the in vivo activities of NPs, it is important to ensure that the radionuclide used for labeling has minimal impact on the original biological activities of NPs.

### CINMs for PAI-guided PTT

PAI has the advantage of high spatial resolution of acoustic imaging and high contrast of optical imaging. It enables in vivo deep tissue imaging by using acoustic waves generated by transient thermoelastic expansions of specific tissues under laser irradiation as the imaging signal [195]. PAI has the potential for precise tumor localization, therapeutic monitoring and in vivo visualization of nanomedicines [97]. The current strategies for enhancing PTT can also be used to enhance PAI, because most PTAs have the potential to be PA contrast agents based on the same photothermal conversion effects and similar photophysical properties [22].

Endogenous chromophores such as melanin and hemoglobin can be utilized for PAI, however, their weak NIR absorption tends to present unsatisfactory contrast. To optimize the imaging effects, it is necessary to develop exogenous contrast agents with high NIR absorption and high stability. A nanosystem (SCP-CS) with excellent photothermal conversion abilities composed of ultrasmall CuS NPs (CS) and semiconducting polymers (SCP) has been reported [192]. Due to its strong NIR absorption abilities, good biocompatibility and photostability, the SCP was used as a PA contrast agent. The strong absorbance of CS components in NIR-I and NIR-II windows potentiated the imaging performance, enabling dual-laser-excited PAI (Fig. 9F, G). SCP-CS exhibited photodynamic as well as chemodynamic efficacy and was validated in vivo.

The rapid development of precision medicine is placing greater demands on imaging technologies for diagnostic and therapeutic procedures. Compared to the “always-on” exogenous contrast agents, the TME-responsive activatable PA reagents exhibit high signal-to-noise ratios and real-time dynamic detection, which can enhance the specificity as well as sensitivity of imaging to accurately localize tumors. Wang et al. developed iron-copper co-doped polyaniline nanoparticles (Fe-Cu@PANI) that could respond to high GSH levels in the TME and induce a red shift of absorption spectrum towards the NIR region via redox reactions, thereby activating PTT and PAI for more accurate PAI-guided PTT in vivo (Fig. 9H–K) [193]. Since the GSH level was related to tumor growth rate and PAI signal correlated with the GSH level, the GSH-responsive PAI contrast agent could monitor tumor growth, providing important information related to disease progression and effectively informing therapy.

### Multimodal imaging-guided PTT

**Fig. 10:**
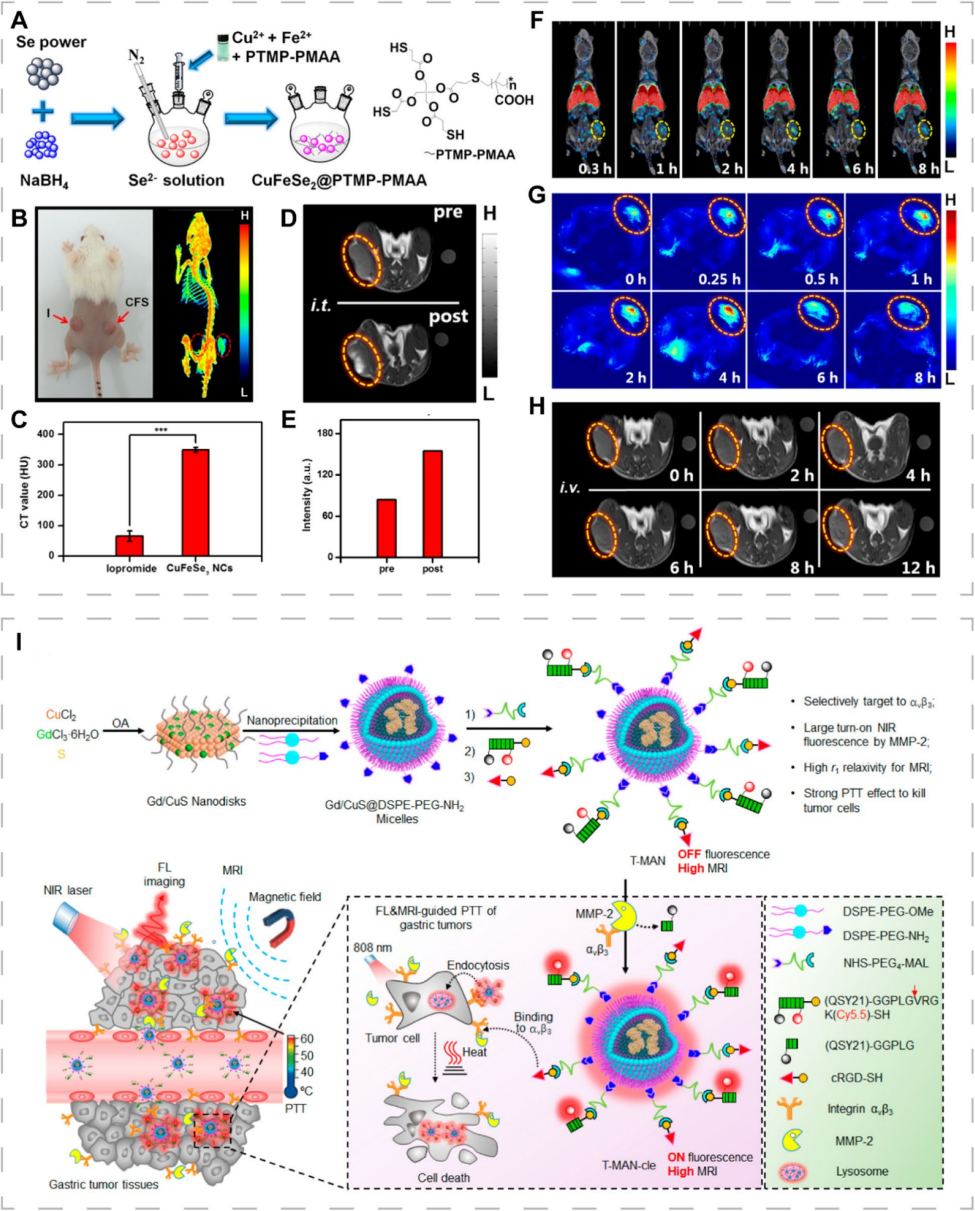
CINMs for multimodal imaging-guided PTT. **A** Synthetic procedures of multifunctional CuFeSe_2_ NCs. **B** Photograph and 3D CT image. **C** CT signal intensity of tumor-bearing mice injected intratumorally with the same concentration of iopromide and CuFeSe_2_ NCs. **D** T_1_WIs and (**E**) corresponding signal intensity of tumor-bearing mice before and after intratumoral injection of CuFeSe_2_ NCs. **F** SPECT images, **G** PAIs and (**H**) T_1_WIs of tumor-bearing mice after intravenous injection of CuFeSe_2_ NCs. Reproduced with permission [21]. Copyright 2017, American Chemical Society. **I** Synthetic procedures and mechanisms of T-MAN. Reproduced with permission [196]. Copyright 2019, American Chemical Society

The multimodal imaging-guided diagnostic and therapeutic platforms have a great potential for overcoming the limitations of single imaging techniques. Jiang et al. prepared radioactive ^99m^Tc-labeled ultrasmall magnetic CuFeSe_2_ NCs with excellent PCE (82%), high X-ray attenuation coefficient, superparamagnetism, good stability and biocompatibility (Fig. 10A) [21]. The NCs integrated PAI, CT, SPECT and MRI in multimodal imaging-guided PTT for precise and comprehensive imaging and treatment (Fig. 10B-H). The good tissue penetration depth and sensitivity of PAI makes up for the limitations of the traditional optical imaging techniques while MRI, CT and SPECT overcome the limitation of low resolution of PAI.

Well-designed multimodal imaging nanoprobes can further improve spatiotemporal precision of imaging and enhance the therapeutic efficacy while reducing phototoxicity to surrounding tissues by adding targeted components and intelligently utilizing various stimuli in the TME. Shi et al. developed an activatable probe (T-MAN) combining tumor receptor-mediated uptake and enzymatic activation strategies [196]. The Gd-doping CuS micellar NPs were modified with NIR fluorophores (Cy5.5), RGD ligands and QSY21-labeled matrix metalloproteinase-2 (MMP-2)-cleavable peptide substrates, where QSY21 acted as a quencher of Cy5.5 and could temporarily turn off fluorescence in non-target tissues. The T-MAN could bind integrin α_v_β_3_ overexpressed on tumor cell membranes and be delivered into gastric tumor tissues. Then, T-MAN was recognized and cleaved by MMP-2 overexpressed in the tumor extracellular matrix, restoring fluorescence and demonstrating excellent fluorescence imaging abilities. Moreover, T-MAN had a good MRI contrast and PCE (70.1%). Validation in gastric MKN45 tumor mice revealed that T-MAN could not only be used as an MRI contrast agent for accurate tumor detection, but also enable near-infrared fluorescence (NIRF) imaging-guided PTT for detection and treatment of primary and metastatic gastric cancer (Fig. 10I). Such imaging probes that can be activated by cancer-associated enzymes and target tumors can be applied to other cancers, and activators in tumor tissues can be accordingly expanded, such as tumor hypoxia, antioxidants, enzymes, and acidic environment.

Multifunctional CINMs that combine diagnostic and therapeutic functions are essential to address the challenge of tumor heterogeneity, paving the way for the goal of personalized precision therapy through image-guided treatment and real-time efficacy monitoring. CINMs with controlled morphology and composition can meet specific imaging and therapeutic needs by integrating targeting and imaging agents. Ingenious design can enhance the aggregation of CINMs in tumor tissues, for improved imaging. Notably, the loading ratios of imaging and therapeutic agents need to be balanced to avoid weakening the function of each modality to ensure long-term performance. .

## Applications of CINMs-based PTT in tissue regeneration

Copper is an essential component and cofactor for proteins and enzymes. Copper affects the expression of intracellular signaling pathways and regulates cellular function, which is essential for tissue repair [57, 197]. For example, copper can stabilize the expression of hypoxia-inducible factor-1α, upregulate vascular endothelial growth factor (VEGF), and promote angiogenesis to provide sufficient oxygen and nutrients for tissue regeneration [6, 198]. Copper also upregulates the expression of osteogenesis-related genes in mesenchymal stem cells, promotes osteogenic differentiation and bone mineral formation, and accelerates bone repair [199]. For tissue infections, copper has been widely used to eliminate bacteria and reduce the risk of poor tissue healing due to infection [6]. In addition, enhancing cell viability through mild PTT is also one of the important ways by which copper promotes tissue regeneration [5]. Here, this section presents the progress of CINMs-based PTT in skin, bone and other organs or tissues (cornea, periodontal tissue, uterus).

### Skin tissue engineering

Wound healing is a complex physiological process that includes hemostasis, inflammation, proliferation and remodeling [200]. Unlike acute wounds that typically heal in an organized manner without significant intervention, chronic wounds often fail to heal promptly, placing a substantial burden on the healthcare system [4]. Although the etiology of chronic wounds varies, they usually share common features, including persistent infection, excessive inflammation, high oxidative stress states, reduced levels of growth factors, impaired angiogenesis, and hypoxia [201, 202]. Therefore, correcting metabolic disturbances in the chronic wound environment by targeting the above factors is very promising. Currently, CINMs-based PTT has been found to upregulate VEGF expression, increase blood supply to the wound site, stimulate the proliferation of fibroblasts, eliminate bacterial infection, and alleviate persistent inflammation, thereby accelerating wound healing [6].

The persistent infection, considered one of the most significant challenges in wound healing, has been extensively discussed. And CINMs-based PTT has been widely used to treat skin wound infections [31, 62, 198]. Specifically, the PTT can safely and effectively combat bacterial infections and antibiotic resistance by interfering with normal bacterial functions, disrupting bacterial integrity, and eradicating bacterial biofilms [60, 203–207]. Even though Cu^2+^ at low concentrations can play a role in bacterial metabolic processes, high Cu^2+^ concentrations can exert their own redox properties, destroying the structure of proteins and nucleic acids and exerting a bactericidal effect [18, 208–210]. Recently, Yang et al. prepared a naturally-derived composite microsphere (Cu^II^-CMC-GelMA/PDA) with good bioadhesion and antibacterial function for infected wound treatment [211]. The copper ions continuously released from the microspheres can synergize with PDA-mediated PTT to exert antibacterial effects, and also accelerate angiogenesis, thus accelerating the healing of *S. aureus-*infected wounds.

**Fig. 11:**
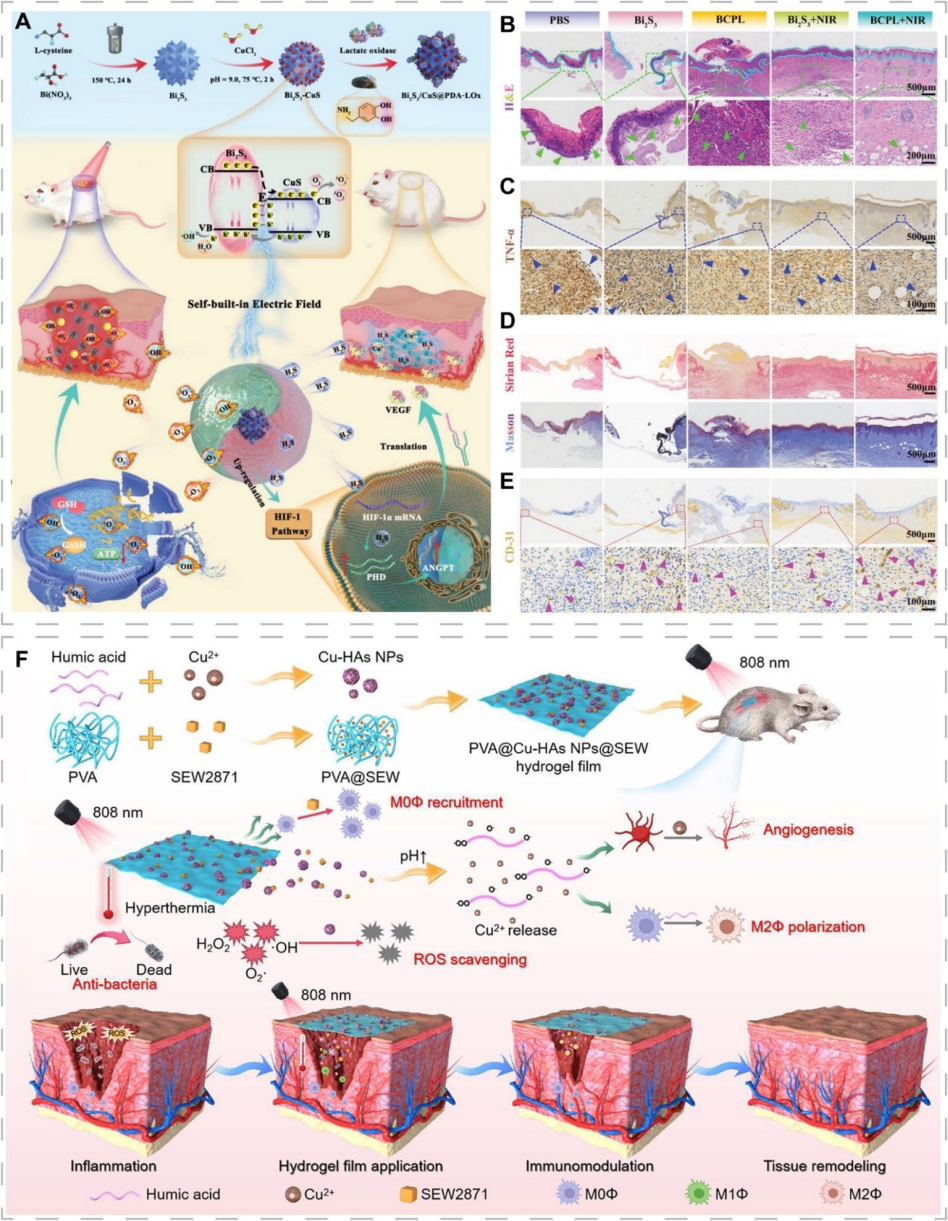
CINMs-based PTT for skin tissue regeneration. **A** Schematic of the synthesis of Bi_2_S_3_/CuS@PDA-LOx and the mechanism for promoting the healing of infected wounds. H&E staining (**B**) and TNF-α staining (**C**) of skin tissue. **D** Sirius red staining and Masson’s trichrome staining of wound tissue to indicate collagen formation. **E** CD31 staining for assessment of angiogenesis in wound tissue after treatment. Reproduced with permission [24]. Copyright 2023, Wiley-VCH. **F** Schematic of the mechanism of NIR/pH dual-responsive PVA@Cu-HAs NPs@SEW films for the treatment of infected wounds. Reproduced with permission [197]. Copyright 2023, American Chemical Society

To achieve the desired therapeutic effect, a higher PTA dose or higher power excitation light is usually required, which increases non-specific thermal damage to the surrounding normal tissues [212]. Designing PTT-based multifunctional antibacterial nanomaterials and combining them with other antibacterial mechanisms, such as PDT and CDT, can improve the antibacterial efficacy and reduce the potential damage to tissues caused by over-reliance on single therapies. Huang et al. prepared a bio-heterojunction (Bi_2_S_3_/CuS@PDA-LOx) that synergized PTT/PDT/CDT against bacteria for the treatment of infected wounds (Fig. 11A) [24]. The combination of n-type bismuth sulfide (n-Bi_2_S_3_) and p-type CuS generated a self-built electric field that enhanced both the antibacterial activity of PTT/PDT and promoted tissue reconstruction. Lactate oxidase (LOx) catalyzed the formation of H_2_O_2_ from lactate in the infected microenvironment, which not only reduced the local lactate concentration, but also provided sufficient substrate for copper-mediated CDT. In addition, the bio-heterojunction was able to release H_2_S in situ, which synergized with the released copper ions to inhibit inflammation and improve blood circulation, thereby accelerating the healing of infected wounds (Fig. 11B-E).

Light, as an external stimulus, can facilitate photothermal therapy and modulate drug delivery in a non-contact, non-invasive, and highly controllable manner, which allows for more precise treatment of wounds [87, 213]. Recently, Yao et al. developed a metal-organic framework microneedle (MN) patch embedded with graphene oxide encapsulated copper-benzene-1,3,5-tricarboxylate NPs (NO@HKUST-1@GO, NHG) [214]. NHG-MN patch achieved photothermal responsive release of nitric oxide under NIR irradiation, accelerating blood vessel formation and collagen deposition to promote diabetic wound healing. Considering that the microenvironments of different types of chronic wounds vary significantly, designing CINMs that are responsive to light and the wound microenvironment, including pH, bacterial toxins, glucose levels, and specific enzymes, will facilitate on-demand wound therapy [201]. It is widely recognized that pH at the wound site is dynamic and correlates with the stage of wound healing, microbial colonization, and other factors. Therefore, pH-responsive CINMs are widely studied [200, 202, 215]. Zha et al. developed a pH-responsive hydrogel membrane (PVA@Cu-HAs NPs@SEW) for healing of infected wounds by utilizing humic acids (HAs), whose solubility is affected by pH, as a copper ion carrier (Fig. 11F) [197]. In the early stage of wound healing, localized thermotherapy of Cu-HAs under NIR irradiation was effective in killing bacteria. Meanwhile, the macrophage-recruiting agent SEW2871 (SEW) recruits M0 macrophages and promotes their polarization toward the M1 phenotype against bacteria. In the later stages of wound healing, elevated pH at the wound site induced the release of copper ions, which promoted angiogenesis. HAs exerted anti-inflammatory and antioxidant functions by promoting M2 macrophage polarization and scavenging ROS, respectively. Ultimately, this hydrogel membrane, which integrated anti-infective, immunomodulatory, ROS scavenging, and angiogenesis modulation functions, effectively promoted the healing of infected wounds.

The current power density required for most CINMs exceeds the maximum skin exposure criterion (0.33 W·cm^−2^ for 808 nm). Unlike tumor treatments, shallower wounds with skin infections usually do not require high power densities of light, which may cause unnecessary damage to surrounding healthy tissue [216]. Therefore, identifying appropriate laser irradiation parameters, including NIR laser wavelength, irradiation duration, and power density, along with the development of sensitive skin temperature monitoring techniques, can help reduce the side effects of CINMs-based PTT and enhance its applicability for skin tissue regeneration.

### Bone tissue engineering

Pathological conditions such as infections, tumors, and trauma can disrupt the integrity of the bone and lead to bone defects. Although bone tissue has the ability to repair itself, larger bone defects require therapeutic intervention. Conventional treatments, including autotransplantation and allogeneic transplantation, are limited due to disadvantages such as limited donor sites and immune rejection reactions [217]. The introduction of bone tissue-engineered scaffolds with good biocompatibility and bioactivity has become an alternative approach, and they can support cell adhesion, proliferation, and differentiation at the site of bone defects [218]. Easily functionalized CINMs can play a synergistic role in bone tissue reconstruction, such as enhancing osteogenic differentiation and angiogenesis, resisting infection, modulating inflammation, and killing invasive bone tumor [219].

Clinically, bone implant-associated infections often lead to failure of bone repair procedures. This is mainly due to bacterial adhesion and colonization followed by biofilm formation [220]. Biofilms block antibiotic penetration as well as diffusion and protect the microorganisms from other external stresses as well as host immune system. Although biofilm can be temporarily removed by surgical debridement, residual biofilm will reemerge and mature within 2–3 days [221]. Therefore, it is necessary to design novel and efficient CINMs to disrupt biofilm and prevent biofilm formation to ensure bone tissue regeneration. Mei et al. prepared a H_2_O_2_-rich biofilm microenvironment-responsive copper-doped polyoxometalate nanoclusters (Cu-POM), which enabled full-stage biofilm removal [222]. Cu-POM interfered with bacterial metabolism via PTT/CDT, leading to bacterial cuproptosis-like death and biofilm disintegration, and activated the immune response of macrophages to enhance chemotaxis and phagocytosis, which resulted in the removal of bacteria from disintegrating biofilms. Preventing biofilm formation is a more rational and effective approach than destroying mature biofilms. Wang et al. reported a TA/Cu-PEG hybrid membrane composed of PEG and tannic acid/Cu^2+^ (TA/Cu) complexes [208]. The inherent antifouling functions of the hydrophilic polymer PEG prevented more than 90% of the initial bacterial adhesion, and TA was involved in constructing the antibacterial surface. Under NIR laser irradiation, Cu^2+^ acted as a photothermal biocide to kill bacteria breaking through the antifouling layer. This strategy of introducing photothermal bactericidal components on antifouling surfaces restricts the initial formation of biofilms and utilizes PTT to kill the few bacteria that break through the hydrated layer, achieving effective and long-lasting antibiofilm effects.

**Fig. 12:**
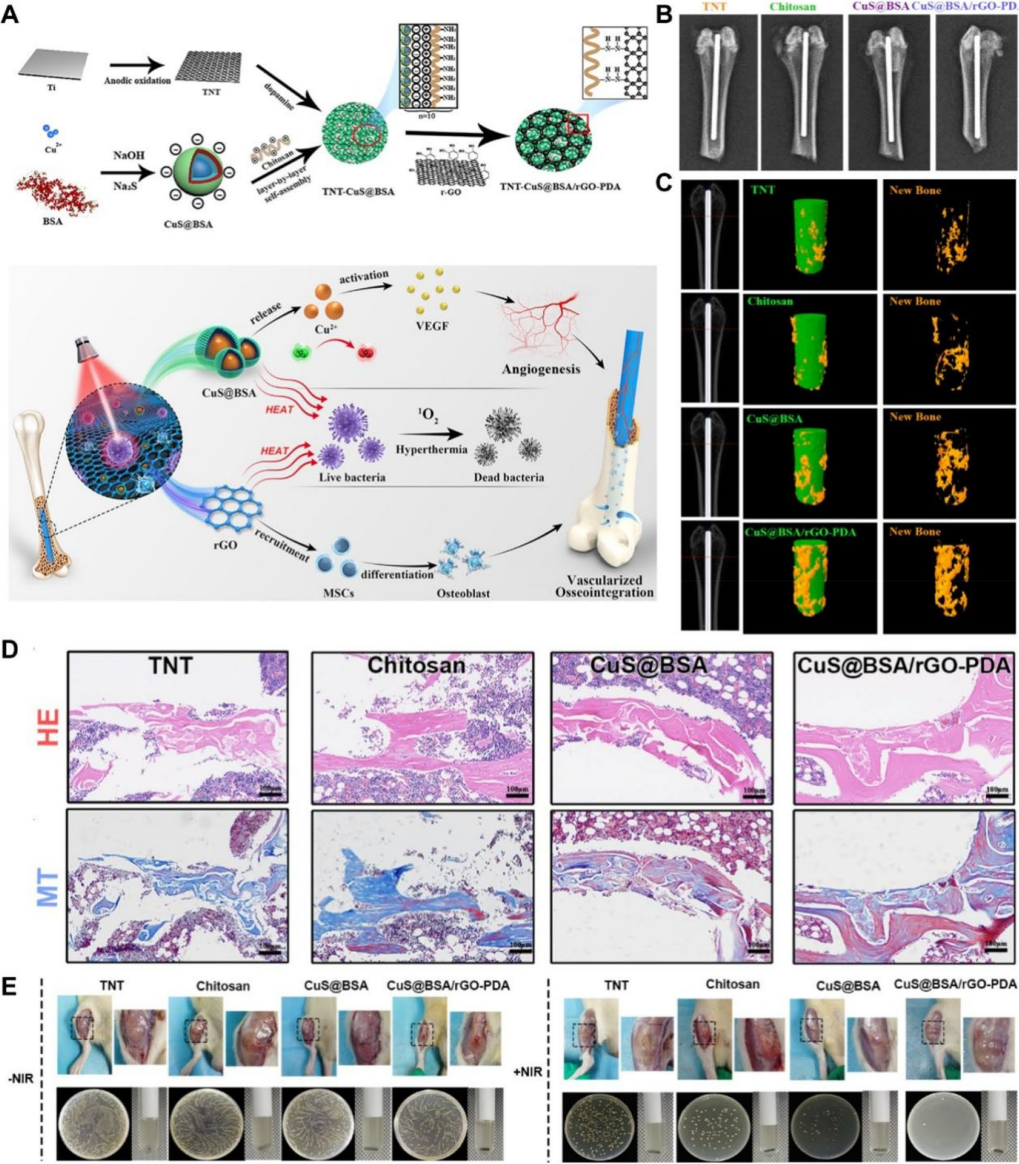
CINMs-based PTT for bone tissue regeneration. **A** Schematic diagram of synthetic and therapeutic mechanisms of CuS@BSA/rGO-PDA for synergistic antibacterial and osseointegration. **B** X-ray examination of intrafemoral implants in rats. **C** Micro-CT scanning of 3D reconstruction of new bone tissues around the implant and no-implant groups at 8 weeks. TNT represents TiO_2_ nanotubes. **D** HE and Masson staining of rat femurs after different treatments. **E** Macroscopic observation of knee-joints after implantation and images of Mueller-Hinton Broth medium and colony plates from different treated posterior rods. Reproduced with permission [224]. Copyright 2021, Elsevier

Thermal stimulation itself promotes migration, proliferation, adhesion and osteogenic differentiation of bone marrow mesenchymal stem cells (BMSCs) and accelerates bone tissue healing [223]. CINMs-based PTT can further enhance osteogenesis and vascularized bone regeneration, which is more promising for bone repair. Zhang et al. reported a dual photothermal coating (CuS@BSA/rGO-PDA) based on CuS and reduced graphene oxide (rGO) [224]. The coating exhibited antibacterial, anti-inflammatory and pro-osteogenic capabilities without relying on growth factors and could be used for the treatment of bone defects following implant infection. Sustained release of Cu^2+^ promoted peri-implant vascularization, and rGO promoted osteogenic differentiation of BMSCs through the mammalian target of rapamycin signaling pathway. Their photothermal effect significantly improved the antibacterial capacity of the coating. The antibacterial and bone repair capabilities were respectively validated in rat tibial condyle and femur models (Fig. 12). This photoreactive coating with osteogenic and antibacterial activity provides a practical strategy for addressing bone implant-associated infections as well as bone regeneration.

Invasion and surgical intervention of bone tumors often result in large bone defects, which are more difficult to repair due to factors such as inadequate blood supply [225]. In addition, residual tumor cells after surgery may lead to tumor recurrence [80]. The development of nanomaterials with dual functions of killing residual tumor cells and promoting bone regeneration is imperative. Dang et al. successfully prepared Cu-TCPP-TCP composite scaffolds by doping copper-ligated tetrakis (4-carboxyphenyl) porphyrin (Cu-TCPP) NSs with good photothermal properties on the surface of 3D printed β-tricalcium phosphate (TCP) scaffolds [199]. The scaffolds were able to kill LM8 osteosarcoma cells by the photothermal effect and released Cu^2+^, which promoted angiogenesis of human umbilical vein endothelial cells and osteogenic differentiation of BMSCs. The scaffolds also released bioactive Ca^2+^ and PO_4_^3−^, which stimulated the expression of osteogenesis-related proteins. In vivo, under NIR irradiation, the Cu-TCPP-TCP scaffolds significantly ablated bone tumors and promoted new bone formation.

### Applications in other organs or tissues

Diabetes-related eye complications, such as diabetic keratopathy and diabetic retinopathy, pose a threat to patients’ vision and affect their quality of life [226]. Qiao et al. prepared AuAgCu_2_O nanoshells consisting of a Cu_2_O shell and hollow gold-silver core for the treatment of nonhealing keratitis. Under NIR irradiation, AuAgCu_2_O nanoshells generated heat and released Cu^+^ and Ag^+^, which acted as an accelerator of lesion healing and antibacterial agent in MRSA-infected mice with diabetic keratitis [227]. Ye et al. developed AuAgCu_2_O-BS NPs using AuAgCu_2_O loaded with bromfenac sodium with anti-inflammatory ability, which could treat post-cataract endophthalmitis by mild PTT-assisted antibacterial and anti-inflammatory effects [228]. The efficacy and safety of AuAgCu_2_O nanogels in combination with PTT in the treatment of severe drug-resistant bacterial keratitis in the human eye is currently under clinical investigation (clinicaltrials.gov id# NCT05268718) .

**Fig. 13:**
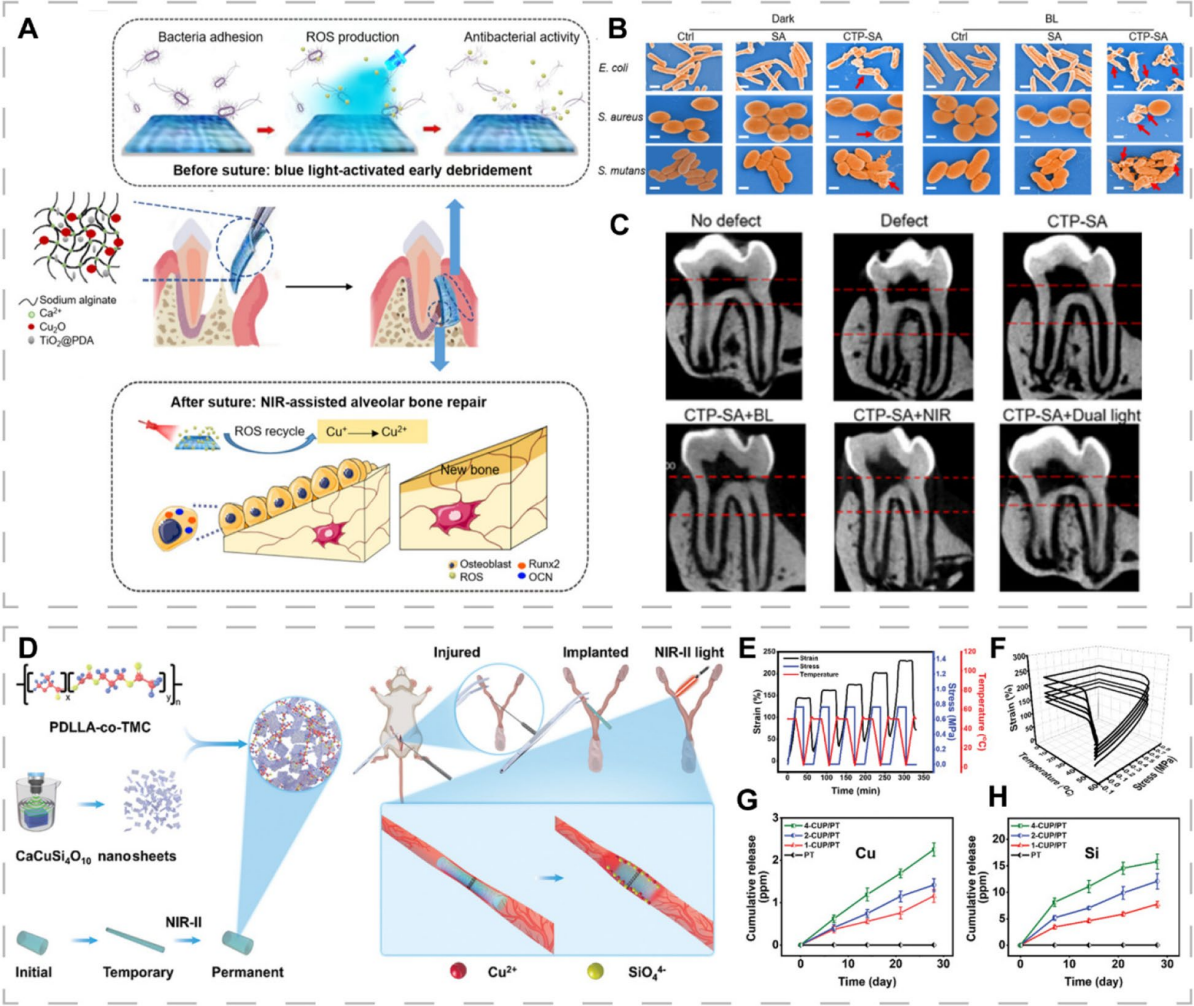
CINMs-based PTT for periodontal tissue and uterus. **A** Schematic illustration of the therapeutic mechanisms of CTP-SA and GTR surgery. **B** SEM images of *Escherichia coli* (*E. coli)*, *S. aureus* and *Streptococcus mutans* after different treatments. **C** Micro-CT images of maxillary first molar of rats in different treatment groups. Reproduced with permission [231]. Copyright 2020, American Chemical Society. **D** Schematic diagram of synthesis of CUP/PT and applications in endometrial regeneration. **E** Two-dimensional and (**F**) 3D shape memory stress-strain-temperature curves of CUP/PT. Cumulative release of (**G**) Cu ions and (**H**) Si ions from different groups. Reproduced with permission [232]. Copyright 2022, Wiley-VCH

Periodontitis, characterized by irreversible damage to both the hard and soft tissues surrounding the teeth, is the most common cause of tooth loss in adults. From both a functional and aesthetic perspective, it can be distressing for patients [229]. Guided tissue regeneration (GTR) is one of the commonly used treatments. Ideal GTR materials should possess excellent antibacterial, osteogenic, biocompatible, and shape-matched properties to the defect site [230]. Additionally, it should be capable of effectively transitioning between antibacterial and osteogenic modes according to the requirements of different healing stages. Xu et al. prepared a dual-light-responsive sodium alginate hydrogel (CTP-SA) by doping Cu_2_O NPs and PDA-modified titanium dioxide (TiO_2_) NPs [231]. Liquid CTP-SA could gel into a solid state after injection to precisely match the defect area. Under blue light excitation, TiO_2_@PDA generated ROS, which could synergize with Cu_2_O NPs, known for their excellent antibacterial properties, to further enhance the antibacterial effect. Additionally, it could oxidize Cu^+^ to Cu^2+^, preparing the ground for the osteogenic phase. Under NIR irradiation, the PTT based on CTP-SA, in synergy with Cu^2+^, promoted osteogenesis. Therefore, CTP-SA with dual-light responsiveness could switch between antibacterial and osteogenic properties through the transformation of copper ion valence states, meeting the requirements of different treatment stages (Fig. 13A-C).

CINMs for uterine endometrial regeneration have also been reported. Endometrial damage caused by infection or mechanical injury can lead to the formation of intrauterine adhesions, increasing the risk of female infertility [233]. Physical anti-adhesion implants such as balloons and intrauterine devices may reduce the occurrence of adhesions by separating the uterine walls, but their ability to promote endometrial healing is limited. Therefore, it is important to develop anti-adhesion implants that can effectively repair the endometrium. Dong et al. prepared NIR-II light-responsive shape memory nanomaterials (CUP/PT) consisting of cuprorivaite (CaCuSi_4_O_10_) NSs and poly(d,l-lactide-*co*-trimethylene carbonate) (PT) for the prevention of intrauterine adhesions and repair of damaged endometrium [232]. PT was a shape-memory polymer that can be fixed to a temporary shape and return to its original shape upon external heat stimulation. CaCuSi_4_O_10_ NSs, as degradable PTAs, generated thermal effects under NIR-II laser irradiation, which could promote the restoration of PT at the site of endometrial injury and exert anti-adhesion effects. They could undergo biodegradation, releasing bioactive copper and silicon ions, thereby promoting vascularization and endometrium repair (Fig. 13D-H).

In conclusion, copper regulates multiple processes in tissue repair, including promoting angiogenesis, stimulating osteogenesis, and exhibiting antibacterial properties. PTT can combat bacteria and tumors and create a favorable microenvironment for tissue healing. CINMs-based PTT combines many advantages and has great potential for tissue regeneration.

## The biosafety of CINMs

Copper, as an essential trace element for living systems, is directly involved in a variety of biological processes such as cell proliferation, neuropeptide synthesis and respiration. The recommended daily dietary intake for adults is 900 μg [33]. Copper homeostasis is essential for cellular metabolism. Abnormal accumulation of intracellular copper can trigger mitochondrial stress leading to cuproptosis [234]. Copper ions are more cytotoxic than iron ions. Fortunately, the delivery of CINMs in the body is not in ionic form, thus effectively reducing their toxicity. Only excess copper causes toxicity, whereas copper that can be excreted from the body does not [68].

The biosafety of CINMs must be thoroughly evaluated before they are actually put into clinical use. The focus of the study should include the toxicity, pharmacokinetics, and pharmacodynamics. The toxicity of CINMs depends on their biological and physicochemical properties, such as dissolution, agglomeration, size, shape, structure, and surface functionality [33]. Copper ions dissolved on the surfaces of CINMs play an important role, leading to membrane rupture, ROS generation, metabolic abnormalities, and cell death [235]. The size, shape and surface functionality of CINMs can influence their intracellular uptake, biodistribution, and clearance. Smaller-sized NPs tend to exhibit higher cellular uptake efficiency, wider biodistribution, and easier renal excretion [136]. CINMs with different morphologies exhibit different circulation time and cellular uptake efficiency [37]. Appropriate surface functionalization avoids nonspecific recognition of CINMs by the immune system, prolongs circulation time, and improves biocompatibility. Common ligands include PEG, proteins, and folic acid [47, 51, 75]. Of note, the mechanisms by which the interactions between the shape, size and surface functionality of CINMs affect toxicity remain unclear. In addition to the intrinsic properties of CINMs described above, exposure factors such as dose, duration of treatment, and mode of administration also influence the toxicity of CINMs [37, 68]. Differences in these exposure factors can lead to different toxicity of the same CINMs.

Recent studies have aimed to elucidate the biosafety of CINMs through in vitro (cell line experiments) and in vivo (animal experiments) experiments. The cytotoxicity of CuO NPs has been studied in human airway epithelial cells (Hep-2), human alveolar basal epithelial cells (A549), human hepatocellular carcinoma (HepG2). These studies suggested that the cytotoxicity of CuO NPs was produced by inducing oxidative stress in a dose- and time-dependent manner [236–238]. Toxicity assessments using murine, fish and worm models also reported that oxidative stress produced by CuO NPs could interfere with hormone levels, neuronal function, liver and kidney function, and immune function. Bioaccumulation of CuO NPs may cause organ toxicity to the brain, kidneys, liver, intestines, stomach and lungs [239]. Considering that toxicity is dose- and time-dependent, the dose of CuO NPs must be strictly controlled to minimize toxicity. Furthermore, Naz et al. noted that biosynthesis using plant extracts could prepare CuO NPs with lower toxicity and higher biocompatibility than chemical or physical synthesis methods [32].

The long-term toxicity of CINMs is of concern due to the poor biodegradability of inorganic materials [240]. Although most NPs can be excreted from the body, the fate and potential effects of NPs retained in the body for a long time remain unknown [38]. Most of the current biosafety assessments of CINMs are relatively short-term (usually less than 1 month), and there is a lack of conclusive evidence on the long-term biosafety. Biodegradable CINMs, such as copper-based metal-organic frameworks, and HCuS NPs, have shown great advantages. Guo et al. reported the biodistribution and degradation process of HCuS NPs in mice, and concluded that HCuS NPs were almost non-toxic at a single dose of 20 mg/kg copper [241]. Intravenously injected HCuS NPs gradually disintegrated into small-sized CuS NPs, and further degraded to copper ions, which were excreted through the hepatobiliary (67%) and renal (23%) within 1 month. After 3 months, no copper residue was found in other organs except spleen and liver. It is worth mentioning that increasing the biodegradability of CINMs may affect their intracellular uptake at the expense of their stability.

The main safety issue in the process of CINMs-based PTT is the lack of thermal restraint mechanism. Therefore, real-time monitoring and regulation of the target tissue temperature is extremely necessary to ensure the therapeutic effect and avoid unnecessary damage to the surrounding tissues due to overheating. Techniques such as PAI, MRI, and diffuse optical tomography have been developed for real-time temperature monitoring of PTT and have shown high sensitivity and specificity [149]. Cao et al. utilized PAI for real-time monitoring of temperature-mediated photoacoustic signal changes in PTT based on polypyrrole@CuS nanohybrid [242]. Histological analyses after in vivo PTT showed a clear boundary between areas with and without NIR irradiation. Apart from thermal damage to adjacent healthy tissues, CINMs-based PTT and photothermal-derived therapies did not cause organ damage or inflammatory lesions in mouse models, nor did they affect blood markers, showing low toxicity in several studies reporting in vivo biosafety assessments [19, 26, 82].

## Discussion and future perspectives

Due to their excellent physicochemical properties and tunable nanostructures, CINMs are promising nanomaterials in the biomedical field. The CINMs-mediated photothermal combination therapy has considerable potential for anti-tumor and accelerated tissue regeneration. This review presents recent advances in CINMs-based PTT for cancer therapy, imaging, and tissue regeneration. Even though significant advances have been made in studies on CINMs, clinical translation remains challenging. The following recommendations are made to address the current issues.

First, a comprehensive long-term assessment of the biosafety of CINMs is essential. The in vivo behaviors of CINMs, including pharmacokinetics and pharmacodynamics are important. Premature release before reaching the target tissue may cause potential toxicity, while prolonged retention in the body may damage organs, including the liver and kidneys. CINMs with high stability are often difficult to degrade in vivo. Possible solutions include performing appropriate surface modifications to improve targeting abilities and biocompatibility, and developing biodegradable CINMs by optimizing their compositions, size and shape. When some non-biodegradable components are introduced to prepare multifunctional diagnostic and therapeutic platforms, their benefits and adverse effects should be critically evaluated. In conclusion, absorption, distribution, metabolism, and excretion processes of CINMs should be assessed through long-term tracking and evaluation to ensure that they meet the quality control standards and safety for new drug development.

Second, the current research focus of preclinical and clinical PTT is disconnected. Clinical research is focused on development of laser devices while preclinical research is focused on preparation of multifunctional PTAs. Most of the high-quality multifunctional CINMs are introduced through complex processes with functional components such as biomolecules and particles, which may result in various challenges in industrial production and limit their clinical applications. The tendency of copper to oxidize in air also limits the large-scale production. The design and preparation of CINMs should be simple, reproducible, economical, and green, with trade-offs between costs and benefits. Attention should be paid to the quantity and purity of raw materials required for the preparation of CINMs. It is important to establish strictly controlled and standardized preparation processes. A comprehensive evaluation system should be established to assess the performance of CINMs, so as to provide a reliable scientific basis for the future development of CINMs. Knowledge and technologies from multiple fields such as bioinformatics, materials science, chemistry, and computer science can be combined to facilitate clinical translation. For example, computational simulations can be used to assess the impact of phototherapy on healthy tissue surrounding the lesion.

Third, preclinical evaluation and clinical implementation of combination therapies are more complex than monotherapies. When designing CINMs for combination therapies, the mechanisms of the various combination therapies and the interactions with complex organisms should be considered. Clarifying the interactions between thermal effects and the microenvironment allows for a more rational and simpler optimization of the nature and function of CINMs. Avoid the simple superposition of cell death-based therapies, as this not only increases systemic toxicity but may also generate new drug resistance. It is important to delve into the signaling pathways and alternative mechanisms involved in the various therapies based on CINMs using multiple analytical and assay methods. In order to maximize the use of each therapy, complementary and synergistic effects of the various therapies should be ensured and their temporal and spatial effects should also be considered. In the case of oncology treatment, it is important to select the appropriate treatment modality and its intensity and duration, and to create programmed treatment modalities based on the status of the tumor at different treatment stages (e.g., tumor distribution and volume). This is cost-effective, that is, optimal efficacy and minimal adverse effects at the lowest dose or intensity, and also allows for personalized treatment for different types of tumors.

Last, the current preclinical animal models cannot accurately predict human therapeutic effects because they cannot replace human clinical features, which limits the clinical translation of CINMs. Development of animal models that better mimic human clinical features may overcome this limitation, especially in functional validation of CINMs involved in immune mechanisms. If possible, large animals such as monkeys or pigs could be used to evaluate the pharmacokinetics and therapeutic effects of CINMs in vivo.

## Conclusion

In conclusion, CINMs are highly competitive and promising nanomaterials for the biomedical field. Their low cost, easily tunable nanostructures and compositions, unique physicochemical properties, and multifunctionality enable them to provide breakthrough platforms for tumor therapy, imaging and tissue regeneration. Various photothermal-derived therapies and smart activation mechanisms based on CINMs have been widely explored in the biomedical field, providing new opportunities to address the challenges of poor efficacy of conventional therapies.

Recent studies have shown that PTT can accelerate ROS production in CDT and PDT, control the release of therapeutic agents in chemotherapy, and accelerate blood supply to alleviate the hypoxic microenvironment, improving the efficacy of other oxygen-dependent therapies. Therefore, PTT combination therapies based on CINMs exhibit excellent ability to kill tumor cells and bacteria. Moreover, CINMs-based PTT can not only increase wound blood supply, stimulate fibroblast proliferation and relieve inflammation to accelerate wound healing, but also promote the proliferation, adhesion and migration of BMSCs, as well as the formation of bone matrix, thereby promoting bone repair. However, in this review of CINMs-based PTT, it is found that the clinical translation of CINMs still faces many challenges, including concerns about their biosafety, difficulties in large-scale preparation, unclear mechanisms of various combination therapies, and inaccurate prediction of human therapeutic efficacy in preclinical animal models. In the future, multi-team and multi-disciplinary collaborations are required to achieve intelligent designs and clinical translation of CINMs. As researchers continue to explore the application potential of CINMs, reveal the complexity of the interactions between CINMs and organisms, and overcome the existing challenges, we can foresee that CINMs-based PTT, as an emerging personalized treatment modality, will play a key role in the biomedical field.

## Data Availability

Not applicable.

## Abbreviations

CINMs: Copper incorporated nanomaterials
PTT: Photothermal therapy
PTAs: Photothermal agents
CDT: Chemodynamic therapy
PDT: Photodynamic therapy
LSPR: Localized surface plasmon resonance
NIR: Near-infrared
PCE: Photothermal conversion efficiency
PAI: Photoacoustic imaging
NSs: Nanosheets
HSPs: Heat shock proteins
MDR: Multidrug resistance
TME: Tumor microenvironment
DDSs: Drug delivery systems
DOX: Doxorubicin
CuS: Copper sulfide
NPs: Nanoparticles
NIR-II: Second near-infrared
RGD: Arginine-glycine-aspartate
P-gp: P-glycoprotein
GSH: Glutathione
ROS: Reactive oxygen species
DSF: Disulfiram
OH: Hydroxyl radicals
PEG: Polyethylene glycol
H_2_O_2_: Hydrogen peroxide
MnO_2_: Manganese dioxide
PDA: Polydopamine
GOx: Glucose oxidase
^1^O_2_: Singlet oxygen
Ce6: Chlorin e6
PET: Positron emission tomography
MHT: Magnetic hyperthermia
TEM: Transmission electron microscopy
PIT: Photoimmunotherapy
TAAs: Tumor-associated antigens
ICD: Immunogenic cell death
CpG ODNs: Cytosine-phosphate-guanine oligodeoxynucleotides
BSA: Bovine serum albumin
DC: Dendritic cell
TIM: Tumor immunosuppressive microenvironment
ICB: Immune checkpoint blockade
PD-1/PD-L1: Programmed cell death protein 1 and its ligands
IDO: Indoleamine-2,3 dioxygenase
RNP: Ribonucleoprotein
3D: Three-dimensional
MRI: Magnetic resonance imaging
CT: Computed tomography
SPECT: Single photon emission computed tomography
T_1_WI: T_1_-weighted imaging
T_2_-FLAIR: T_2_-weighted fluid-attenuated inversion recovery imaging
NIRF: Near-infrared fluorescence
NCs: Nanocrystals
MRSA: Methicillin-resistant *Staphylococcus aureus*
rGO: Reduced graphene oxide
BMSCs: Bone marrow mesenchymal stem cells
MN: Microneedle
GTR: Guided tissue regeneration
